# The Optimal Exercise Modality and Dose for Cortisol Reduction in Psychological Distress: A Systematic Review and Network Meta-Analysis

**DOI:** 10.3390/sports13120415

**Published:** 2025-11-24

**Authors:** Xiongjie Li, Jianping Huang, Feilong Zhu

**Affiliations:** 1 School of Physical Education, Wuhan Sports University, Wuhan 430079, China; lixiongjie0314@163.com (X.L.); hjp0724@163.com (J.H.); 2 School of Sports Medicine and Rehabilitation, Beijing Sport University, Beijing 100084, China

**Keywords:** psychological disorder, cortisol reduction, exercise intervention, dose–response, network meta-analysis

## Abstract

**Objectives**: Psychological distress has been linked to dysregulation of the hypothalamic–pituitary–adrenal (HPA) axis and altered cortisol secretion. Exercise is increasingly recognized as a non-pharmacologic strategy for stress regulation. This systematic review and network meta-analysis assessed the relative efficacy of different exercise modalities and the optimal dose in modulating cortisol levels in adults with psychological distress. **Methods**: We systematically searched five databases up to 30 June 2025 for relevant randomized controlled trials. Two reviewers independently conducted study selection, data extraction, and risk-of-bias assessments. Pairwise meta-analyses and a frequentist network meta-analysis were performed with random-effects models. Standardized mean differences (SMDs) with 95% confidence intervals (CIs) were calculated. Dose–response relationships were examined, and the certainty of evidence was evaluated using the GRADE framework. **Results**: Forty-four studies were included. Overall, exercise was associated with moderate cortisol reductions. Yoga demonstrated the greatest effect (SMD = −0.59; 95% CI = −0.90 to −0.28; SUCRA = 93%), followed by qigong and multicomponent exercise. High-intensity interval training tended to increase cortisol levels, although not significantly. The dose–response analysis revealed an inverted U-shaped relationship, characterized by an optimal response at approximately 530 MET-min/week, and longer intervention duration predicted greater reductions. The certainty of evidence ranged from very low to high, with yoga–control comparisons supported by the strongest evidence. **Conclusions**: Exercise, particularly mind–body practices such as yoga and qigong, can reduce cortisol levels in individuals with psychological distress. The dose–response relationship exhibited a non-linear pattern, with optimal efficacy observed at approximately 530 MET-min/week. Nevertheless, further high-quality trials are required to confirm the optimal modality and dose.

## 1. Introduction

Psychological distress refers to the negative emotional responses elicited by exposure to stressors [1], with anxiety, depression, and stress being its core manifestations [2]. In the absence of effective coping mechanisms, persistent psychological distress is associated with an elevated risk of severe psychiatric disorders and broad impairments in both physical and mental health [3]. Epidemiological studies provide strong support for these associations. The Global Burden of Disease study in 2021 reported that the age-standardized incidence rate of mental disorders increased by 15.2% from 1990 to 2021. Supporting these findings, a 3-year follow-up cohort study found that individuals with high psychological distress had a 3.4–4.0-fold higher risk of developing the first major depressive episode compared with those reporting low distress [4]. These findings highlight that sustained psychological distress, particularly in the absence of adequate regulatory mechanisms, plays a key role in both the onset and rising incidence of psychiatric disorders. 

Dysregulation of the hypothalamic–pituitary–adrenal (HPA) axis is widely recognized as a core mechanism linking psychological distress with altered cortisol regulation [5]. In depression, anxiety, and chronic stress, the HPA axis is frequently hyperactivated, resulting in abnormal secretion of cortisol [6]. Early or acute phases tend to be marked by hyperactivation and sustained cortisol elevation, whereas prolonged stress exposure may lead to a blunted or exhausted hormonal response [7]. These contrasting profiles, which manifest as either elevated basal cortisol or an impaired cortisol awakening response (CAR), exemplify the dynamic and non-linear nature of HPA axis adaptation. Although cortisol plays an essential role in energy mobilization and acute stress adaptation, its chronic dysregulation leads to deleterious long-term consequences [8]. According to the Glucocorticoid Cascade Hypothesis, persistent dysregulation of cortisol levels initiates a cascade of detrimental processes, resulting in cumulative damage such as neuronal injury, amplified inflammatory responses, and disruption of circadian rhythms [9].

Pharmacologic therapy is the primary approach to cortisol regulation in clinical settings, most often with selective serotonin reuptake inhibitors (e.g., fluoxetine) or glucocorticoid agents (e.g., hydrocortisone) [10,11]. Prolonged use, however, may reduce effectiveness and increase the risk of adverse effects, limiting its role in long-term management [12]. Exercise intervention, in contrast, provides a noninvasive alternative that may lower costs while conferring additional benefits for both physical and mental health [13]. Different effects have been observed across various types of exercise. Mind–body interventions, including yoga and qigong, are generally linked to reductions in cortisol, most likely through dampening physiological arousal and enhancing parasympathetic tone [14]. Findings regarding conventional aerobic exercise (e.g., brisk walking, cycling) has yielded inconsistent results: some studies demonstrate meaningful cortisol reductions [15], whereas others report negligible changes or even counterintuitive increases, particularly at higher intensities [16]. Evidence for resistance training remains relatively limited, yet available studies [17] suggest its effects are dose-dependent, adding further complexity to interpretation. Substantial methodological heterogeneity limits the comparability of existing studies. First, study populations differ markedly, ranging from clinically diagnosed patients to subclinical or otherwise healthy individuals under stress. Second, key intervention parameters, such as intensity, frequency, session duration, and overall program length vary considerably across trials, complicating the identification of an optimal dosage. Finally, the current evidence base relies predominantly on small-scale randomized controlled trials (RCTs) with heterogeneous designs and insufficient statistical power, which precludes the formulation of definitive clinical guidelines. 

Although several narrative reviews have discussed the potential of exercise to modulate cortisol and alleviate psychological distress, only a few quantitative syntheses have been conducted. Beserra et al. [18] reported that regular exercise reduced daytime cortisol in individuals with depression, but their findings were limited by heterogeneous sampling protocols and small sample sizes. Anderson and Wideman [19] reviewed studies on the cortisol awakening response and found inconsistent evidence, largely due to methodological variability. Hayes et al. [20] demonstrated that acute exercise alters salivary cortisol in men, yet most data concerned short-term effects in healthy participants. Collectively, these reviews suggest that exercise can influence cortisol regulation, but uncertainties remain regarding the optimal modality, intensity, and dose, particularly in psychologically distressed populations. To address these gaps, we conduct a systematic review and network meta-analysis (NMA) to compare the effects of different exercise modalities and doses on cortisol levels, providing evidence to inform individualized exercise prescriptions.

## 2. Method

### 2.1. Registration and Protocol

The review adhered to the Preferred Reporting Items for Systematic Reviews and Meta-Analyses (PRISMA) guidelines [21]. The protocol was prospectively registered with PROSPERO (CRD420251084343).

### 2.2. Search Strategy 

A comprehensive literature search was performed using the electronic databases PubMed, PsycINFO, Scopus, Web of Science, and Embase from their inception until June 30, 2025. The search strategy employed a combination of controlled vocabulary (e.g., Medical Subject Headings [MeSH]) and free-text terms related to “psychological distress,” “exercise therapy,” “physical activity,” “exercise intervention,” and “randomized controlled trial.” The full search strategy is detailed in Supplementary Material S1.

Beyond electronic database searches, we additionally reviewed reference lists of prior systematic reviews to capture further eligible studies. To reduce selection bias, two reviewers (X.L. and J.H.) independently assessed titles and abstracts of all retrieved records, with subsequent full-text evaluation of potentially relevant articles. Inter-rater agreement was quantified using Cohen’s kappa, interpreted against established benchmarks [22]. Disagreements were resolved through consensus or, when necessary, adjudication by a third reviewer (F.Z.).

### 2.3. Eligibility Criteria 

The inclusion criteria were formulated in accordance with the PICOS framework: Population (P): Individuals with psychological distress, operationalized as elevated symptoms measured by validated scales (e.g., Beck Depression Inventory [BDI], Depression Anxiety Stress Scales [DASS], or State-Trait Anxiety Inventory [STAI]). This inclusive criterion was adopted to capture a broad spectrum of psychological stress states and enhance the generalizability of our findings. No restrictions were placed on age or gender. Intervention (I): Structured exercise interventions (e.g., aerobic, resistance, or mind–body exercises) performed regularly for a minimum duration of ≥3 weeks. This threshold was selected to ensure the interventions length were sufficient to elicit stable physiological adaptations in the HPA axis and sustainable psychological benefits, rather than capturing acute effects. There were no restrictions regarding intensity, frequency, or modality. Comparison (C): Control groups receiving waitlist, no intervention, usual care, or alternative exercise groups involving different exercise types. Outcomes (O): Studies were required to report changes in cortisol levels, measured using at least one validated biomarker (e.g., salivary, serum, or hair cortisol). The criterion of "at least one" sample was chosen to maximize the inclusivity of the evidence base. Cortisol levels were required to be expressed in standard units such as nmol/L or μg/dL [23]. Study design: Only RCTs were incorporated.

The exclusion criteria are as follows:

(1) Studies involving combined interventions, such as exercise alongside pharmacotherapy or psychological treatment;
(2) Insufficient or non-extractable outcome data for quantitative synthesis;
(3) Non-original research, including reviews, protocols, case reports, conference abstracts, and editorials.

### 2.4. Data Collection and Processing

#### 2.4.1. Data Extraction

Two reviewers (X.L. and J.H.) independently extracted data using a standardized form. From each eligible study, the following data were extracted: study characteristics (first author, publication year, country, design, and sample size); participant details (age, sex, and other relevant demographics); and intervention parameters (exercise modality, frequency, intensity, duration, and total volume). Intervention reporting followed the Template for Intervention Description and Replication (TIDieR) framework [24]; Primary outcomes: pre- and post-intervention cortisol levels, including the type of biological sample (e.g., blood, saliva, hair) and units of measurement (e.g., mmol/L, µg/dL). For studies reporting multiple sampling time points, we prioritized the extraction of morning cortisol levels to enhance comparability. If morning samples were not available, the sampling time was recorded, and this variability was acknowledged as a potential source of heterogeneity.

When effect sizes were not directly provided, we derived them from available summary statistics (e.g., means, standard deviations) following procedures recommended by the Cochrane Handbook [25]. Efforts were made to obtain missing data by contacting corresponding authors where necessary. Any discrepancies between reviewers were resolved through consensus by consultation with a third reviewer (F.Z.). If a study reported multiple outcome measures, the most validated or commonly used metric was selected for analysis.

#### 2.4.2. Data Coding and Management

To ensure analytical consistency, interventions were classified into five categories in Supplementary Material S2: (1) continuous aerobic exercise (e.g., walking) (CAE), (2) yoga, (3) qigong, (4) high-intensity interval training (HIIT), and (5) multicomponent exercise (MCE), which integrated both aerobic and resistance elements. As resistance training was seldom implemented in isolation, it was included only within multicomponent interventions. The overall exercise dose was calculated by multiplying intensity (in metabolic equivalents [METs]), frequency (sessions per week), and duration (minutes per session), and reported as MET-minutes per week (MET-min/week) [26].

Frequency referred to the mean number of sessions conducted weekly throughout the intervention. Duration indicated the active time per session, excluding warm-up and cool-down periods unless specifically included. Exercise intensity was classified based on the 2024 Adult Compendium of Physical Activities [27], a reference providing standardized MET values for numerous physical activities. For studies employing progressive protocols or lacking explicit session duration, the average duration over the entire intervention was estimated.

For the purpose of enhancing comparability and ensuring adequate connectivity within the network meta-analysis model, weekly MET-min/week values were stratified into five discrete categories: 0 (control), 250, 500, 750 and 1000 METs-min/week [28].

### 2.5. Risk of Bias Assessment and Certainty of Evidence 

The methodological quality of the included RCTs was independently assessed by two reviewers (X.L. and J.H.) using the Cochrane Risk of Bias 2.0 tool (RoB 2) [29]. This instrument evaluates five domains: randomization procedures, deviations from intended interventions, incomplete outcome data, outcome measurement, and selective reporting. Assessments were performed at the final post-intervention time point to capture sustained effects. Each domain, as well as the overall judgment, was categorized as “low risk,” “some concerns,” or “high risk.” Any disagreements were resolved through discussion, with arbitration by a third reviewer (F.Z.) when necessary.

The certainty of evidence for each outcome was appraised with the GRADE framework (Grading of Recommendations, Assessment, Development, and Evaluation) [30]. Given that all included studies were RCTs, the baseline level of evidence was rated as “high.” Evidence could be downgraded on the basis of five prespecified factors: risk of bias, inconsistency, indirectness, imprecision, and publication bias. Two reviewers conducted these evaluations independently, and consensus was reached to address any discrepancies. The certainty ratings were classified as high, moderate, low, or very low.

### 2.6. Data Synthesis

All statistical analyses were performed using R software (version 4.3.1). Pairwise meta-analyses were performed with the metafor and meta packages [31], network meta-analysis with netmeta [32], dose–response modeling with MBNMAdose [33], and data visualization with ggplot2 [34].

#### 2.6.1. Pairwise Meta-Analysis

Pairwise meta-analyses were conducted using a random-effects model, with results expressed as standardized mean differences (SMD) and 95% confidence interval (CI) [35]. The degree of statistical heterogeneity was examined using the I^2^ statistic, where values exceeding 50% were considered to represent substantial heterogeneity [36]. Between-study variance was quantified using the τ^2^ statistic [37].

#### 2.6.2. Network Meta-Analysis

We performed a network meta-analysis within a frequentist framework to compare and rank exercise interventions for cortisol outcomes. The analysis was conducted with the netmeta package, which combines direct and indirect evidence [32]. Transitivity was assessed by examining potential effect modifiers across interventions [38]. Consistency was tested globally with the design-by-treatment interaction model and locally with node-splitting. Inconsistency was considered present when the 95% CI excluded zero or when the *p* value was <0.05 [39]. Ranking of interventions was based on surface under the cumulative ranking curve (SUCRA) values [40]. Rankograms and cumulative probability plots were used to visualize the likelihood of each intervention being the most effective. 

#### 2.6.3. Dose–Response Network Meta-Analysis

We also conducted a dose–response NMA using a model-based framework (MBNMA) to assess the relation between exercise dose and treatment effects. Exercise dose was expressed as metabolic equivalent task minutes per week using standard conversion methods. Nonlinear dose–response associations were modeled with polynomial functions and restricted cubic splines. Model fit was evaluated with the deviance information criterion (DIC) in Supplementary Material S6, residual deviance, and between-study variability [33]. Based on the best-fitting model, we plotted an overall dose–response curve and separate curves for intervention types. For clinical interpretation, following the Cochrane Handbook (Chapter 15.5.3.3) [25], an SMD of 0.5 [41] was assumed to correspond to a baseline remission rate of 30–50%, equivalent to an absolute improvement of about 15–25%. This provided a pragmatic estimate of clinically meaningful benefit in the absence of a defined minimum clinically important difference (MCID).

#### 2.6.4. Additional and Sensitivity Analyses

To assess the robustness and generalizability of the findings, sensitivity analyses were performed by excluding individual trials and studies with a high risk of bias. Subgroup analyses were conducted by intervention setting. Network heterogeneity was reported using τ^2^ and I^2^. When at least 10 studies were available, publication bias was examined with Egger’s test and funnel plots.

## 3. Result

### 3.1. Studies Included and Characteristics

**Figure 1:**
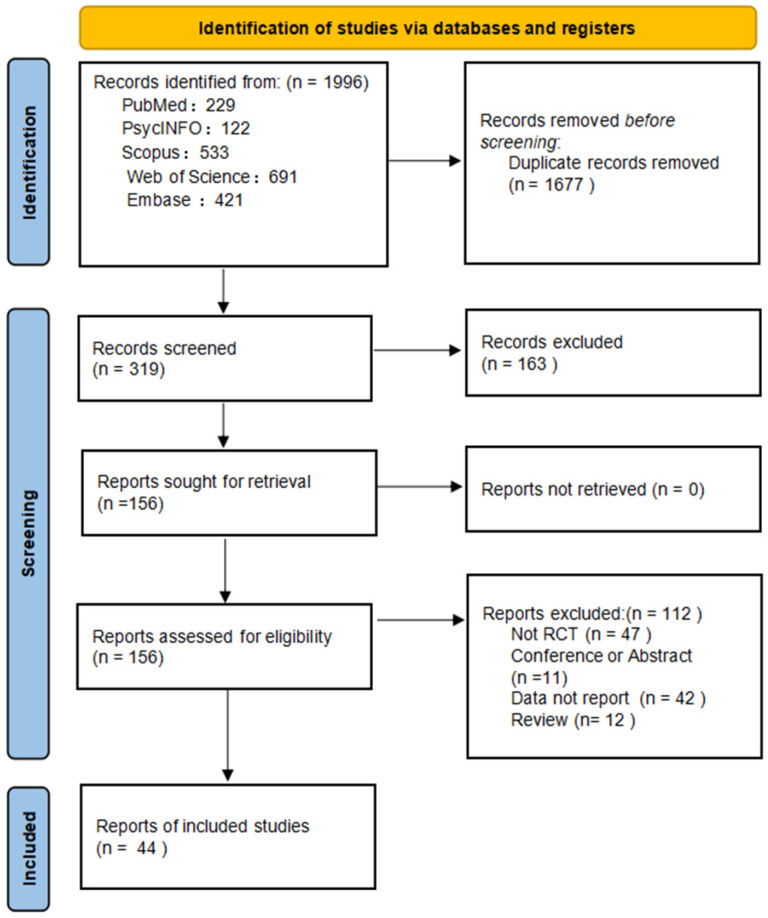
PRISMA flow diagram of study selection.

A total of 1996 records were identified across five databases. After removing duplicates and screening titles and abstracts, 319 articles were assessed in full text. Of these, 44 met inclusion criteria and were included in the analysis (Figure 1). Interrater reliability was high, with k = 0.70 for title/abstract screening and k = 0.80 for full-text screening (Supplementary Material S3).

The included studies, published between 2003 and 2025, involved 3284 participants with psychological distress. Yoga was the most frequent intervention (*n* = 20), followed by continuous aerobic exercise (*n* = 14). On average, interventions lasted 12 weeks, with 3 sessions per week of approximately 55 min each. Study details, including sample size, participant demographics, intervention type, and outcome measures, are provided in Supplementary Material S4.

### 3.2. Pairwise Meta-Analysis

Compared with control, all interventions except HIIT (SMD = 0.53; 95% CI= 0.09 to 0.97; I^2^ = 23.46%) were associated with reductions in cortisol concentration, although not all reductions reached statistical significance. Among the significant results, yoga (SMD = −0.67; 95%CI = −1.08 to −0.25; I^2^ = 90.86%) showed the largest average reduction, while multicomponent exercise showed smaller effects ((SMD = −0.01; 95%CI = −0.21 to 0.19; I^2^ = 0%). Pairwise meta-analysis results are provided in Supplementary Material S5.1.

### 3.3. Network Meta Analysis

**Figure 2:**
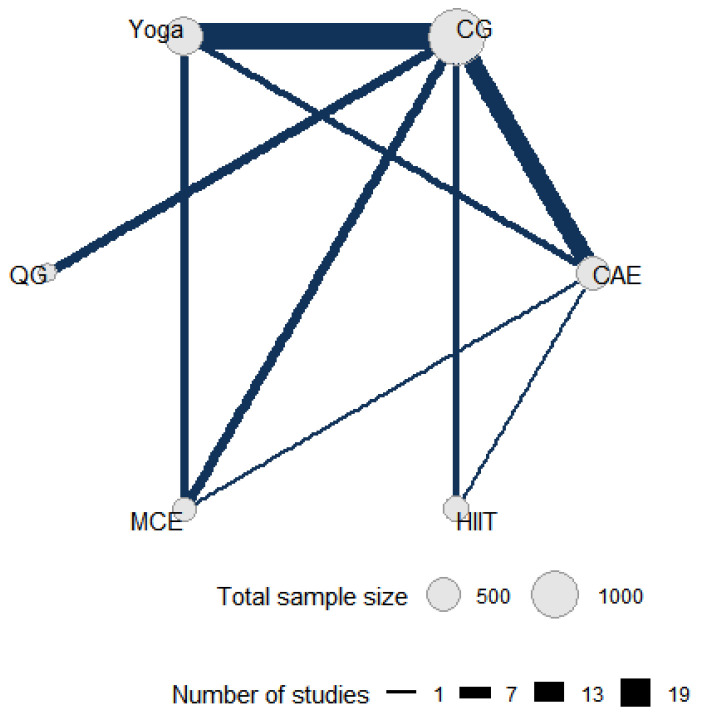
Direct and indirect comparisons in a network meta-analysis. CG: control group; HIIT: high-intensity interval training; MCE: multicomponent exercise; QG: qigong; CAE: continuous aerobic exercise.

Figure 2 shows the direct comparative relationship among the five intervention measures included in this network meta-analysis, namely yoga, control group, qigong, multicomponent exercise, aerobic exercise and high-intensity interval training. In the network diagram, each node represents an intervention, and its size is proportional to the total sample size of patients allocated to that intervention. The connections between nodes represent studies with direct comparisons, and the number of studies with direct comparisons directly correlates with the thickness of the connections.

A frequentist network meta-analysis was conducted, including 48 studies and enabling both direct and indirect comparisons across all exercise (Supplementary Material S5.3). Yoga ranked highest in cortisol-lowering efficacy (SMD = −0.59; 95% CI = −0.90 to −0.28), followed by qigong (SMD = −0.42; 95% CI = −1.03 to 0.20). Both direct and indirect evidence were included in the network meta-analysis, the point estimate for HIIT indicated a potential increase in cortisol levels; however, the confidence intervals was wide and now included the null value (SMD = 0.23; 95% CI = −0.47 to 0.94). This discrepancy with the pairwise meta-analysis is likely due to the incorporation of indirect comparisons, which introduced greater uncertainty and modified the effect estimate. 

**Figure 3:**
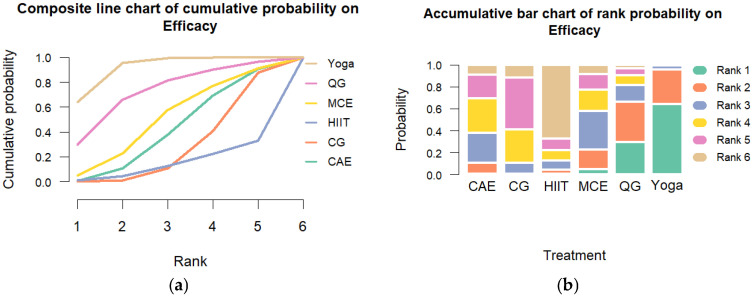
Cumulative Ranking Probability Plot of Exercise Interventions. Note: (**a**) Composite line chart of cumulative probability on Efficacy. (**b**) Accumulative bar chart of rank probability on Efficacy.CG: control group; HIIT: high-intensity interval training; MCE: multicomponent exercise; QG: qigong; CAE: continuous aerobic exercise.

Transitivity was considered acceptable based on comparable distributions of age, baseline cortisol levels, and intervention duration across study arms. Global consistency assessment using the design-by-treatment interaction model indicated significant inconsistency (Q = 32.22, df = 13, *p* = 0.002). Heterogeneity was substantial (τ^2^ = 0.448; I^2^ = 86.3%; 95% CI: 82.6% to 89.3%). Node-splitting analyses revealed no significant inconsistency in most pairwise comparisons (*p* > 0.05), enhancing the model’s robustness. The SUCRA rankings indicated that yoga had the highest likelihood of being the most effective intervention (SUCRA = 93%), followed by qigong (SUCRA = 73%) and multicomponent exercise (SUCRA = 52%). Rankograms and cumulative probability plots are presented in Figure 3.

### 3.4. Dose–Response Relationship

**Figure 4:**
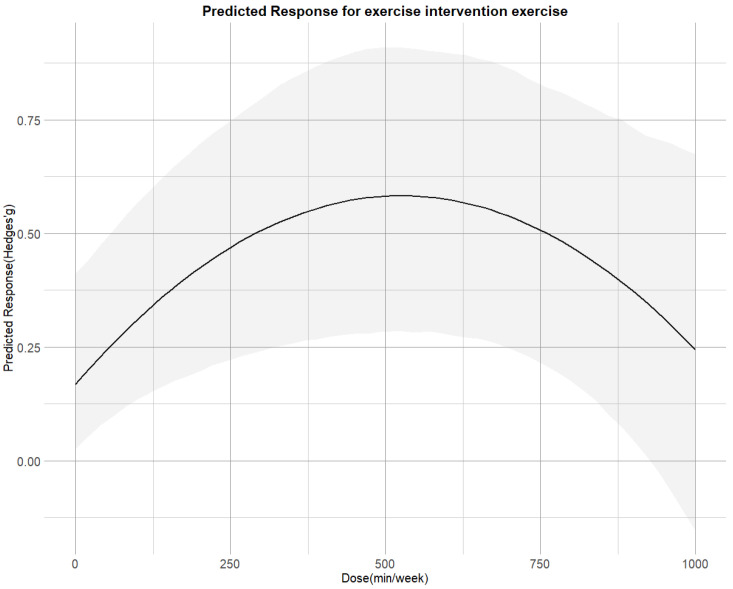
Dose–response associations between exercise doses and improving cortisol.

Figure 4 illustrates that the non-linear dose–response relationship was found for the entire range of exercise doses. The overall curve exhibited an inverted U-shape, with cortisol reduction increasing up to 530 METs-min/week (SMD = 0.58;95% CI = 0.28, 0.92), beyond which the effect plateaued. The predicted dose corresponding to the MCID was approximately 300 METs-min/week.

**Figure 5:**
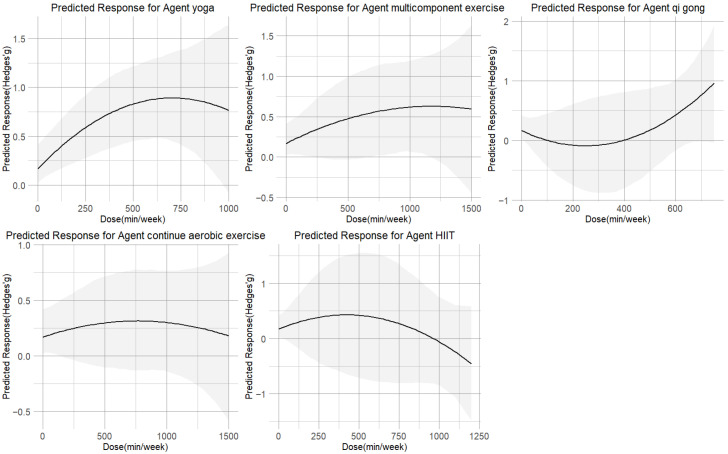
Dose–response associations between different types of exercise doses and improving cortisol.

The dose–response relationships for each of the five exercise modalities are shown in Figure 5. The inverted U-shaped curve for yoga peaked at 630 METs-min/week (SMD = 0.85; 95% CI = 0.48 to 1.25; SD = 0.19). Multicomponent exercise (peak at 1200 METs-min/week, SMD = 0.60, 95% CI = −0.01 to 1.22; SD = 0.31) showed curves that plateaued beyond their respective peak doses. In contrast, qigong demonstrated a U-shaped relationship, reaching its minimal effect (SMD = 0.50; 95% CI = −0.06 to 1.18; SD = 0.31) at approximately 530 METs-min/week. The dose–response relationship for both CAE and HIIT followed an inverted U-shaped curve. However, their profiles differed: CAE reached its peak effect (SMD = 0.31; 95% CI = −0.11 to 0.76; SD = 0.22) at a moderate dose of 770 METs-min/week, with a gradual decline thereafter. HIIT, while showing a potential for reduction at lower doses of 360 METs-min/week, exhibited a much steeper and more pronounced negative response at higher intensities, ultimately resulting in a weaker overall effect that failed to reach a moderate effect size (SMD = 0.32; 95% CI = −0.56 to 1.22; SD = 0.46). Yoga at 650 METs-min/week had the highest chance of being the most successful intervention, according to ranking analysis. Furthermore, Table 1 provides practical recommendations for each exercise type based on the minimal effective dose predictions.

### 3.5. Additional Analysis 

Meta-regression analyses indicated that intervention duration significantly moderated treatment effects (β = −0.03 per week; *p* = 0.04), with longer interventions associated with greater cortisol reductions (Supplementary Material S5.6). In contrast, age, year of publication, and baseline cortisol concentrations were not significant moderators.

Consistent results were observed in sensitivity analyses restricted to studies with lower risk of bias. Egger’s test was not significant (*p* > 0.10), and funnel plots showed no apparent asymmetry, suggesting minimal risk of publication bias (Supplementary Material S5.2). Subgroup analyses revealed that moderate-intensity exercise (3–6 METs) produced a significant reduction in cortisol (SMD = 0.72, 95% CI =0.32 to 1.13), comparable to low-intensity exercise (<3 METs; SMD = 0.77, 95% CI = 0.36 to 1.18), whereas high-intensity exercise (>6 METs) showed a smaller effect (SMD = 0.31, 95% CI = 0.05 to 0.57). Regarding duration, sessions lasting 30–60 min yielded a significant decrease (SMD = 0.57, 95% CI = 0.27 to 1.26), and frequencies of more than three sessions per week showed the greatest benefit (SMD = 1.03, 95% CI = 0.26 to 1.79). 

By symptom category, exercise demonstrated the strongest effect on stress (SMD = 0.90, 95% CI = 0.36 to 1.43), followed by anxiety (SMD = 0.74, 95% CI = −0.01 to 1.50) and depression (SMD = 0.54, 95% CI to 0.22, 0.86).

Across measurement types, significant reductions were observed in salivary cortisol (SMD = 0.58, 95% CI = 0.16 to 1.00) and blood cortisol (SMD = 0.51, 95% CI = 0.32 to 0.69), while hair cortisol showed minimal change (SMD = 0.01, 95% CI = −0.19 to 0.22).

### 3.6. Risk of Bias

Eight trials were classified as having high risk of bias. A detailed risk-of-bias profile is presented in Supplementary Material S9. Sensitivity analyses limited to low-risk studies produced results aligned with the main analysis, reinforcing the robustness of the estimated optimal exercise dose of 540 METs-min per week (Supplementary Material S10).

### 3.7. Certainty of Evidence

The GRADE method was used to grade the degree of evidence certainty, which ranged from very low to high. Because of their indirectness and imprecision, the majority of comparisons were downgraded to low certainty. Only the comparison between yoga and the control reached a high level of certainty; however, due to inconsistency, the comparison between HIIT and the control was downgraded to very low certainty. Supplementary Material S5.5 contains the comprehensive scoring criteria.

## 4. Discussion

### 4.1. Comparative Efficacy of Exercise Types and Underlying Mechanisms

Our findings indicate that exercise interventions showed a tendency towards reducing cortisol levels associated with psychological distress, although this effect was not statistically significant for most modalities. Notably, yoga demonstrated a significant reduction in cortisol. Further ranking using network meta-analysis identified yoga as the most effective intervention, with the highest SUCRA ranking (93%), followed by qigong, MCE, CAE, and HIIT. While previous meta-analyses [18,42,43] have established the general effect of exercise on cortisol, they have typically treated ‘exercise’ as a homogenous intervention. This approach is exemplified by a recent meta-analysis [44], which found a small but significant overall association between physical activity and a steeper diurnal cortisol slope, yet reported substantial heterogeneity and inconclusive results for moderators like activity type and intensity, underscoring the limitations of aggregating disparate exercise forms. Our study extends this literature by directly comparing the effectiveness of various exercise modalities and delineating their distinct dose–response relationships.

The findings from both pairwise and network meta-analyses suggest that yoga significantly reduces cortisol concentration in people with psychological distress (SMD = −0.59; 95% CI = −0.90 to −0.28; SUCRA = 93%). Previous meta-analyses [18] have similarly demonstrated that yoga can effectively reduce the cortisol concentration of people with psychological distress. The efficacy of yoga may stem from its dual action on neuroendocrine regulation: it enhances negative feedback sensitivity of the HPA axis, thereby reducing resting cortisol levels, while its emphasis on breathwork and mindfulness directly stimulates parasympathetic activity, promoting physiological calm [45]. From the perspective of physiological mechanism, systematic yoga training may promote more direct vagus nerve stimulation and the improvement of heart rate variability (HRV) through breathing control and the coupling of posture and breathing [46], which will help maintain cortisol at the baseline level. Qigong, which demonstrated a U-shaped dose–response curve, likely exerts its effects through similar autonomic mechanisms. However, its unique dose–response pattern suggests that there may be 530 MET-min/week for maximizing parasympathetic activation, beyond which additional practice may not yield further cortisol reduction.

In addition to yoga and qigong, MCE, CAE, and HIIT ranked next in terms of efficacy. These interventions were classified as integrated aerobic exercise, which is characterized by aerobic energy supply. Such Aerobic exercise modulates immune–inflammatory signaling by refining the dynamic release of interleukin-6 (IL-6) [47], which subsequently promotes a rapid increase in brain-derived neurotrophic factor (BDNF). Concurrently, aerobic exercise enhances glucocorticoid receptor (GR), which mediated negative feedback within HPA axis [48], thereby attenuating cortisol secretion. The meta-analysis [49] demonstrated that HIIT was generally associated with increased cortisol levels, suggesting that high-intensity exercise may promote cortisol secretion through activation of the HPA axis. Importantly, increased cortisol does not necessarily indicate adverse effects; rather, it may represent an adaptive physiological response to intensive load. Notably, several included randomized controlled trials reported that although HIIT increased cortisol levels, it concurrently alleviated psychological distress [50]. Such mental health benefits may be attributable to potential mechanisms including enhanced BDNF expression, improved neuroplasticity, and regulation of emotional processing.

The subgroup analyses provide important insights into how exercise parameters influence cortisol regulation and psychological outcomes. Moderate-intensity exercise (3–6 METs) appeared most effective, supporting the notion that moderate workloads optimize HPA axis adaptation without triggering excessive physiological stress. In contrast, high-intensity exercise yielded smaller cortisol reductions, possibly due to acute stress responses that temporarily elevate cortisol secretion. Consistent with prior findings, sessions lasting 30–60 min and performed more than three times per week produced robust improvements, highlighting the importance of sustained, regular engagement rather than sporadic high-dose activity.

Symptom-specific effects suggest that exercise may particularly benefit stress regulation (SMD = 0.90), likely through restoring HPA axis responsiveness and enhancing coping capacity. The more modest effects on anxiety and depression might reflect the involvement of additional neurobiological mechanisms—such as monoamine regulation or neuroplastic changes—that interact with cortisol but are not solely determined by it. Overall, these findings underscore that moderate, regular exercise exerts the most reliable benefits on stress-related cortisol dysregulation, offering practical guidance for clinical and behavioral interventions.

### 4.2. Dose–Response Effects Across Exercise Types

Significant differences were observed in the optimal dose ranges of various exercise modalities on cortisol regulation, which may reflect distinct patterns of activation of the HPA axis and the autonomic nervous system. Both yoga and continuous aerobic exercise demonstrated dose–response curves that peaked at moderate levels (approximately 300 and 500 METs-week, respectively), with diminishing effects at higher loads. The mechanisms are consistent with low-dose yoga practices, in which breathing control and meditation enhance parasympathetic activity, and promote neuroplasticity in the hippocampus and prefrontal cortex, alongside modulation of neurotransmitters such as γ-aminobutyric acid (GABA) and serotonin [51], thereby buffering stress responses. Continuous aerobic exercise shows a similar U-shaped pattern: while acute sessions may transiently elevate cortisol, regular training at moderate doses supports restoration of diurnal rhythms and attenuation of chronic stress reactivity. Excessive loads, however, may induce metabolic and cardiovascular strain that counteracts these benefits.

In contrast, multicomponent exercise exhibited a relatively flat curve (maximum at approximately 560 METs-week), suggesting limited direct effects on cortisol. This may be attributable to opposing influences of aerobic and resistance components, with resistance training acutely elevating cortisol and aerobic exercise reducing baseline levels over time [17]. Nonetheless, such combined interventions may indirectly benefit psychological outcomes through improvements in physical fitness and self-efficacy [52], even if cortisol modulation is modest. HIIT revealed a distinct inverted U-shaped response. While low-to-moderate doses were associated with favorable cortisol regulation, higher training volumes resulted in reduced or even negative responses. Mechanistically, HIIT causes the HPA axis and sympathetic nervous system to become rapidly activated, which results in sudden spikes in cortisol levels [53]; however, excessive training frequency or intensity may precipitate HPA axis fatigue or exaggerated negative feedback [54], ultimately blunting cortisol output.

By contrast, qigong demonstrated a continuously ascending dose–response curve, suggesting sustained benefits at higher doses. As a mind–body practice, qigong appears to suppress HPA axis activity, enhance parasympathetic tone, and reduce adrenocorticotropic hormone (ACTH) and cortisol levels [14]. Unlike high-intensity modalities, qigong maintained positive effects even at higher volumes. Prior studies indicate that single sessions can transiently reduce cortisol, whereas regular practice (≥12 weeks) consolidates these effects, improving mood and quality of life [55].

In summary, our results suggest that yoga and qigong exert favorable effects on cortisol regulation even at moderate or higher doses, whereas HIIT and aerobic exercise demonstrate dose-sensitive responses, with excessive loads potentially detrimental. Multicomponent exercise exerts more modest direct effects but may contribute to long-term psychological health through complementary mechanisms. These results highlight the importance of tailoring exercise prescriptions to individual stress regulation capacities and physiological adaptations in populations with psychological distress.

### 4.3. Clinical Implications

First, the present findings reveal that the effect of physical exercise on cortisol levels in people with psychological distress is not linearly dose-dependent but rather follows a non-linear pattern. Our dose–response analysis indicated an inverted U-shaped relationship for total exercise dose, with the maximum benefit observed at approximately 530 MET-min/week. This optimal range aligns with the lower limit of the World Health Organization (WHO) recommendation of 150–300 min of moderate-intensity activity per week (equivalent to 500–1000 MET-min/week), providing a physiological rationale for these public health guidelines. Beyond this threshold, the effect plateaued or declined, suggesting that higher doses do not necessarily confer greater reductions in cortisol and, in some cases, may be counterproductive. This underscores the importance of prescribing exercise at an appropriate dose rather than simply increasing intensity or volume.

Second, both the type of exercise and its optimal dosage are critical considerations. Yoga demonstrated the most robust and clinically meaningful reductions in cortisol, with the biggest impact being seen at about 630 MET-min/week. Qigong showed similarly favorable effects, reaching optimal benefits around 530 MET-min/week, while multicomponent exercise achieved moderate efficacy at approximately 640 MET-min/week. Approximately 770 MET-min per week of continuous aerobic exercise was required to approach a peak, whereas high-intensity interval training did not achieve a clinically meaningful reduction within the tested dose range. These results suggest that mind–body practices should be prioritized as first-line non-pharmacologic interventions, while multicomponent and continuous aerobic exercise may serve as alternative options when appropriately dosed. Conversely, HIIT should be prescribed cautiously, as higher intensities were associated with cortisol elevations.

Finally, given that long-term adherence is essential, clinicians should consider patient preferences, baseline fitness, and tolerability when recommending exercise. Mind–body interventions may be particularly suitable for individuals with lower fitness levels, comorbidities, or poor tolerance for vigorous activity. Meanwhile, structured aerobic or resistance-based regimens may still provide indirect psychological benefits through improvements in physical capacity, sleep quality, and self-efficacy, even if their direct impact on cortisol is less pronounced.

### 4.4. Limitations and Future Directions

First, although our network meta-analysis included 44 randomized controlled trials, the overall sample size within specific exercise modalities was relatively moderate (Supplementary Material S4), limiting the precision of effect estimates. Second, considerable between-study heterogeneity and inconsistency (Supplementary Material S6) were observed, partly attributable to variations in intervention protocols (e.g., session frequency, intensity, duration), participant characteristics, and outcome assessment methods. Third, the measurement of cortisol concentration varied across studies (Supplementary Material S8), with some relying on single-point serum or salivary samples rather than standardized diurnal profiles, which may have introduced measurement bias. Furthermore, the inconsistent reporting of precise cortisol sampling times across included studies prevented a systematic adjustment for diurnal variation, which represents a potential source of measurement heterogeneity. Fourth, while yoga and qigong demonstrated consistent benefits, most evidence originated from studies of moderate methodological quality, and the evidence certainty was rated as low for numerous outcomes. Finally, psychological distress is a multifaceted construct, and cortisol reduction represents only one of its biological correlates; therefore, these findings must be considered alongside broader clinical outcomes like quality of life, functional recovery, and symptom relief.

Future research should prioritize large-scale, multicenter RCTs with standardized intervention protocols and rigorous cortisol sampling methods (e.g., multiple daily assessments across several days). Further exploration of mechanistic pathways including neuroendocrine, inflammatory, and neuroplasticity-related biomarkers will help clarify how exercise influences stress regulation. Additionally, research should evaluate long-term sustainability and adherence, as well as real-world effectiveness in diverse populations. Comparative effectiveness trials directly contrasting mind–body practices with conventional aerobic or resistance training would provide stronger evidence for clinical decision-making. Finally, integrating digital health tools and personalized exercise prescriptions may enhance intervention adherence and optimize therapeutic outcomes for individuals with psychological distress.

## 5. Conclusions

This systematic review and network meta-analysis evaluates and compares the influence of different forms of exercise on cortisol levels among individuals with psychological distress by integrating direct and indirect data. Overall, exercise interventions were linked to moderate cortisol reductions. Yoga provided the most consistent and clinically meaningful benefits, followed by qigong and multicomponent exercise. The dose–response analysis revealed a non-linear pattern, with the greatest cortisol reduction observed at approximately 530 MET-min/week, which is equivalent to 90–150 min of moderate-intensity exercise per week. This finding suggests that optimizing the exercise dosage is more important than merely increasing training intensity. 

These findings highlight exercise, particularly mind–body exercise practices, as an effective non-pharmacological therapeutic intervention for modulating stress physiology in populations with psychological distress. Tailored exercise prescriptions that consider modality, dosage, and individual patient characteristics may optimize both biological and psychological outcomes. Further high-quality RCTs are required to establish evidence-based protocols for implementing these tailored interventions in clinical and public health settings.

## Figures and Tables

**Table 1:** Exercise Recommendations for Improving psychological distress.

Type	Recommend (METs-min/Week)	Intensity	Energy Expenditure (METs-Min)	Recommended Accumulation (Minutes/Week)		Exercise Prescription Suggestion (Sessions x Minutes/Week)	
				Minimum	Optimal	Minimum	Optimal
Overall	300–530	Low	3.5 (code 02034)	90	180	3x~30	4x~45
						6x~15	6x~30
		Moderate	5.0 (code 02035)	60	120	3x~20	4x~30
						6x~15	6x~20
		Vigorous	7.5 (code 02040)	45	90	3x~15	3x~30
						5x~10	6x~15
Yoga	180–630	Low	2.3 (code 02150)	80	270	4x~20	4x~70
						5x~16	6x~45
Qigong	520	Moderate	3.3 (code 15670)	150		5x~30	
						6x~25	
Multicomponent exercise	640	Moderate	5.0 (code 02035)	120		4x~30	
						6x~20	
		Vigorous	7.5 (code 02040)	90		3x~30	
						6x~15	

## Data Availability

The original contributions presented in the study are included in the article/Supplementary Materials. Further inquiries can be directed to the corresponding author.

## Supplementary Materials

The following supporting information can be downloaded at https://www.mdpi.com/article/10.3390/sports13120415/s1. Supplementary Materials S1–S12, Figure S1: Funnel plot of all studies; Figure S2: Dot splitting method to explore inconsistency; Figure S3: Heat map; Figure S4: Treatment-level network; Figure S5: “Split” NMA of overall exercise; Figure S6: “Split” NMA of different exercise agents; Figure S7: Deviance plot at overall exercise level; Figure S8: Deviance plots at treatment-level; Figure S9: Node-splitting analysis (density plot); Figure S10: Subgroup analysis; Figure S11: Study-level of Risk bias Analysis; Figure S12: Dose–response curve between exercise and improves in psychological distress only including studies with low risk of bias; Figure S13: Effectiveness ranking by exercise treatments; Table S1: Definition for term; Table S2: Excluding studies by reading the title and abstract; Table S3: Excluding studies by reading the full text; Table S4: Details of pairwise meta-analyses; Table S5: SUCRA table for all studies; Table S6: GRADE assessment for all pairwise comparisons; Table S7: Model fit summaries for univariate network meta-regression; Table S8: Consistent and UME models fit comparison; Table S9: Comparison of transitivity; Table S10: Models Fit Comparison For Overall Exercise; Table S11: Models fit Comparison For Different Type Exercise; Table S12: Protocol deviations.

## Abbreviations

The following abbreviations are used in this manuscript:

ACTH	Adrenocorticotropic hormone
BDI	Beck Depression Inventory
BDNF	brain-derived neurotrophic factor
CAE	Continuous aerobic exercise
CAR	Cortisol awakening response
CG	Control group
CIs	Confidence intervals
DASSs	Depression Anxiety Stress Scales
DIC	Deviance information criterion
GABA	γ-aminobutyric acid
GR	Glucocorticoid receptor
HIIT	High-intensity interval training
HPA	Hypothalamic–pituitary–adrenal
HRV	Heart rate variability
MCE	Multicomponent exercise
MCID	Minimum clinically important difference
METs	Metabolic equivalents
NMA	Network meta-analysis
PRISMA	Preferred Reporting Items for Systematic Reviews and Meta-Analyses
QG	Qigong
RCTs	Randomized controlled trials
SMDs	Standardized mean differences
STAI	State–Trait Anxiety Inventory
SUCRA	Surface under the cumulative ranking curve
TIDieR	Template for Intervention Description and Replication
WHO	World Health Organization
