# Supplementary material for: The Optimal Exercise Modality and Dose for Cortisol Reduction in Psychological Distress: A Systematic Review and Network Meta-Analysis
Source: Sports (Basel). 2025 Nov 24;13(12):415. doi: 10.3390/sports13120415 (PMC12736704; doi:10.3390/sports13120415)
Supplement: Supplementary file 1 [file sports-13-00415-s001.zip › sports-3942844-supplementary.pdf]

# Optimal Exercise Modality and Dose for Cortisol Reduction in Psychological Distress: A Systematic Review and Network Meta-Analysis

**Table of Contents**

Supplementary Material S1:Search strategy.....1
Supplementary Material S2: Defination.....7
Supplementary Material S3: Kappa score for selecting studies between the reviewers.8
Supplementary Material S4: Dataset.....9
Supplementary Material S5: Network meta- analysis supplementary.....14
S5.1 Details of pairwise meta-analyses.....14
S5.2 Comparison funnel plot of all studies.....14
S5.3 Dot splitting method to explore inconsistency.....15
S5.4 Rank.....16
S5.4.1 SUCRA table for all studies.....16
S5.4.2 Heat map.....16
S5.5 GRADE Assessment.....17
S5.6 Meta-regression.....18
Supplementary Material S6: Dose network meta- analysis supplementary.....19
S6.1:Key assumptions of Network Meta-Analysis.....19
S6.1.1:Connectivity.....19
S6.1.2:Consistency.....20
S6.1.3:Transitivity.....21
S6.2 Models' selection.....24
S6.2.1. Nonlinear functions and models fit comparison.....24
Supplementary Material S7:Ranking of effectiveness of interventions.....27
Supplementary Material S8: Subgroup analysis.....28
Supplementary Material S9: Risk bias Analysis.....29
Supplementary Material S10: Sensitivity analysis including only studies with low...30
Supplementary Material S11:Ranking of effectiveness of interventions.....31
Supplementary Material S12:Updated protocol.....32

## Supplementary Material S1:Search strategy

| | |
|---|---|
| MEDLINE (PubMed) | 1.(("Stress, Psychological"[Mesh] OR "Anxiety"[Mesh] OR "Anxiety Disorders"[Mesh:NoExp] OR "Depression"[Mesh] OR "Depressive Disorder"[Mesh] OR "Burnout, Psychological"[Mesh] OR "Mental Fatigue"[Mesh]) OR ("psychological distress"[tiab] OR "mental distress"[tiab] OR "emotional distress"[tiab] OR "subjective distress"[tiab] OR "perceived stress"[tiab] OR "chronic stress"[tiab] OR "occupational stress"[tiab] OR "work-related stress"[tiab] OR anxiet*[tiab] OR panic[tiab] OR phobi*[tiab] OR depress*[tiab] OR dysthymi*[tiab] OR "affective symptom*"[tiab] OR "emotional symptom*"[tiab] OR "mental fatigue"[tiab] OR burnout[tiab] OR "well-being"[tiab] OR "quality of life"[tiab]))<br>2.(("Exercise Therapy"[Mesh] OR "Exercise"[Mesh] OR "Exercise Movement Techniques"[Mesh] OR "Sports"[Mesh] OR "Yoga"[Mesh] OR "Tai Ji"[Mesh] OR "Pilates-Based Exercises"[Mesh] OR "Resistance Training"[Mesh] OR "High-Intensity Interval Training"[Mesh] OR "Aerobic Exercises"[Mesh] OR "Physical Exertion"[Mesh] OR "Motor Activity"[Mesh])OR (("physical activit*"[tiab] OR exercise[tiab] OR exercises[tiab] OR "physical training"[tiab] OR "fitness training"[tiab] OR "workout"[tiab] OR "sport*"[tiab] OR "athletic*"[tiab]) AND ("aerobic"[tiab] OR "cardio*"[tiab] OR "endurance"[tiab] OR "resistance"[tiab] OR "strength"[tiab] OR "weight training"[tiab] OR "interval training"[tiab] OR "HIIT"[tiab] OR "circuit training"[tiab] OR "flexibility"[tiab] OR "balance training"[tiab] OR "coordination"[tiab] OR "yoga"[tiab] OR "tai chi"[tiab] OR "qigong"[tiab] OR "pilates"[tiab] OR "mind-body"[tiab] OR "martial art*"[tiab] OR "open skill*"[tiab] OR "closed skill*"[tiab] OR "gross motor"[tiab])) OR ("dose"[tiab] OR "dosage"[tiab] OR "dose-response"[tiab] OR "frequency"[tiab] OR "intensity"[tiab] OR "duration"[tiab] OR "volume"[tiab] OR "modality"[tiab] OR "type"[tiab] OR "prescription"[tiab] OR "protocol"[tiab] OR "program*"[tiab] OR "regimen"[tiab] OR "intervention"[tiab] OR "therap*"[tiab]))<br>3.(("Hydrocortisone"[Mesh] OR "Cortisol"[Mesh] OR "Salivary Cortisol"[Mesh]) OR (cortisol[tiab] OR hydrocortisone[tiab] OR "salivary cortisol"[tiab] OR "serum cortisol"[tiab] OR "plasma cortisol"[tiab] OR "blood cortisol"[tiab] OR "hair cortisol"[tiab] OR "urinary cortisol"[tiab] OR "cortisol level*"[tiab] OR "cortisol concentration*"[tiab] OR "cortisol output"[tiab] OR "cortisol secretion"[tiab] OR "cortisol response"[tiab] OR "cortisol |

| | |
|---|---|
| | reactivity"[tiab] OR "cortisol awakening response"[tiab] OR "CAR"[tiab] OR "HPA axis"[tiab] OR "hypothalamic pituitary adrenal"[tiab]))<br>4.(("Randomized Controlled Trial"[pt] OR "Controlled Clinical Trial"[pt] OR "Clinical Trial, Phase III"[pt]) OR (randomi?ed[tiab] OR randomly[tiab] OR randomised[tiab] OR randomized[tiab] OR trial[tiab] OR groups[tiab] OR rct[tiab] OR "random allocation"[mh] OR "random allocation"[tiab] OR "random sequence"[tiab] OR "randomly assigned"[tiab] OR "random assignment"[tiab] OR "random* control*"[tiab] OR "control group"[tiab] OR "controlled trial"[tiab] OR "clinical trial"[tiab])) NOT (animals[mh] NOT humans[mh])<br>#1 AND #2 AND #3 AND #4 |
| Web of Science | #1: TS=(("psychological distress" OR "mental distress" OR "emotional distress" OR "perceived stress" OR anxiet* OR panic OR phobi* OR depress* OR dysthymi* OR "affective symptom*" OR burnout OR "mental fatigue" OR "well-being" OR "quality of life") NOT ("post-traumatic" OR "posttraumatic" OR ptsd OR "combat disorder*"))<br>#2: TS=((("physical activit*" OR exercise OR exercises OR "physical training" OR fitness OR workout OR sport* OR athletic*) AND (aerobic OR cardio* OR endurance OR resistance OR strength OR "weight training" OR "interval training" OR HIIT OR "circuit training" OR flexibility OR "balance training" OR coordination OR yoga OR "tai chi" OR qigong OR pilates OR "mind-body" OR "martial art*" OR "open skill*" OR "closed skill*" OR "gross motor")) OR "dose" OR dosage OR "dose-response" OR frequency OR intensity OR duration OR volume OR modality OR type OR prescription OR protocol OR program* OR regimen OR intervention OR therap*)<br>#3: TS=(cortisol OR hydrocortisone OR "salivary cortisol" OR "serum cortisol" OR "plasma cortisol" OR "blood cortisol" OR "hair cortisol" OR "urinary cortisol" OR "cortisol level*" OR "cortisol concentration*" OR "cortisol response" OR "HPA axis" OR "hypothalamic pituitary adrenal")<br>#4: TS=(randomi?ed OR randomly OR randomised OR randomized OR trial OR groups OR rct OR "random allocation" OR "randomly assigned" OR "random assignment" OR "random* control*" OR "control group" OR "controlled trial" OR "clinical trial") |

| | |
|---|---|
| | #5: #1 AND #2 AND #3 AND #4 |
| Scopus | (<br>TITLE-ABS-KEY ( "psychological distress" OR "mental distress" OR "emotional distress" OR "perceived stress" OR anxiet* OR panic OR phobi* OR depress* OR dysthymi* OR "affective symptom*" OR burnout OR "mental fatigue" )<br>AND<br>TITLE-ABS-KEY ( ( ( "physical activit*" OR exercise OR exercises OR "physical training" OR fitness OR workout OR sport* ) W/3 ( aerobic OR cardio* OR endurance OR resistance OR strength OR "weight training" OR "interval training" OR HIIT OR yoga OR "tai chi" OR qigong OR pilates OR "mind-body" OR "martial art*" ) ) OR dose OR dosage OR "dose-response" OR frequency OR intensity OR duration OR volume OR modality OR type OR prescription OR protocol OR program* OR regimen OR intervention OR therap* )<br>AND<br>TITLE-ABS-KEY ( cortisol OR hydrocortisone OR "salivary cortisol" OR "serum cortisol" OR "plasma cortisol" OR "blood cortisol" OR "hair cortisol" OR "cortisol level*" OR "cortisol response" OR "HPA axis" )<br>AND<br>TITLE-ABS-KEY ( randomi?ed OR randomly OR randomised OR randomized OR rct OR "random allocation" OR "randomly assigned" OR "random assignment" OR "controlled trial" )<br>)<br>AND ( LIMIT-TO ( DOCTYPE , "ar" ) OR LIMIT-TO ( DOCTYPE , "cp" ) )<br>AND ( LIMIT-TO ( LANGUAGE , "English" ) ) |
| Embase | ((TI ( ("psychological distress" OR "mental distress" OR "perceived stress" OR anxiet* OR depress* OR burnout) ) OR AB ( ("psychological distress" OR "mental distress" OR "perceived stress" OR anxiet* OR depress* OR burnout) ) OR MW ("Stress" OR "Anxiety" OR "Depression"))<br>AND<br>((TI ( ( (exercise OR "physical activit*" OR training OR sport*) N3 (aerobic OR resistance OR strength OR interval OR HIIT OR yoga OR "tai chi" OR pilates) ) OR (dose OR dosage OR intensity OR frequency OR duration) ) OR AB ( ( (exercise OR "physical |

| | |
|---|---|
| | activit*" OR training OR sport*) N3 (aerobic OR resistance OR strength OR interval OR HIIT OR yoga OR "tai chi" OR pilates) ) OR (dose OR dosage OR intensity OR frequency OR duration) ) OR MW ("Exercise" OR "Exercise Therapy" OR "Physical Activity" OR "Sports"))<br>AND<br>((TI ( cortisol OR hydrocortisone OR "salivary cortisol" OR "HPA axis" ) OR AB ( cortisol OR hydrocortisone OR "salivary cortisol" OR "HPA axis" ) OR MW ("Cortisol"))<br>AND<br>((TI ( randomi?ed OR randomly OR rct OR "random allocation" OR "controlled trial" ) OR AB ( randomi?ed OR randomly OR rct OR "random allocation" OR "controlled trial" ) OR MW ("Randomized Controlled Trials"))) |
| PsycINFO | 1. exp "Stress (Psychological)"/ or exp Anxiety/ or exp "Anxiety Disorders"/ or exp Depression/ or exp "Depressive Disorders"/ or exp "Burnout"/ or exp "Mental Fatigue"/<br>2. ("psychological distress" or "mental distress" or "emotional distress" or "perceived stress" or anxiet* or panic or phobi* or depress* or dysthymi* or "affective symptom*" or burnout or "mental fatigue").ti,ab,id.<br>3. 1 or 2<br>4. exp "Exercise"/ or exp "Physical Activity"/ or exp "Sports"/ or exp "Yoga"/ or exp "Tai Chi"/ or exp "Aerobic Exercise"/ or exp "Strength Training"/<br>5. (("physical activit*" or exercise or exercises or "physical training" or fitness or workout or sport*) adj3 (aerobic or cardio* or endurance or resistance or strength or "weight training" or "interval training" or HIIT or yoga or "tai chi" or qigong or pilates or "mind-body" or "martial art*")).ti,ab,id.<br>6. (dose or dosage or "dose-response" or frequency or intensity or duration or volume or modality or type or prescription or protocol or program* or regimen or intervention or therap*).ti,ab,id.<br>7. 4 or 5 or 6<br>8. exp "Hydrocortisone"/ or exp "Cortisol"/<br>9. (cortisol or hydrocortisone or "salivary cortisol" or "serum cortisol" or "plasma cortisol" or "blood cortisol" or "hair cortisol" or "cortisol level*" or "cortisol response" or "HPA axis").ti,ab,id.<br>10. 8 or 9<br>11. exp "Randomized Controlled Trials"/ |

| | |
|---|---|
| | 12. (randomi?ed or randomly or randomised or randomized or rct or "random allocation" or "randomly assigned" or "random assignment" or "controlled trial").ti,ab,id.<br>13. 11 or 12<br>14. 3 and 7 and 10 and 13<br>15. limit 14 to human |

**Supplementary Material S2: Defination**

**Table S1.** Definition for term

| Name | Definitions |
|---|---|
| Yoga | Yoga is a mind-body practice that integrates physical postures, stretching exercises, controlled breathing, and meditative focus to enhance flexibility, muscle tone, relaxation, and mental well-being. The concept specifically refers to yoga practice emphasizing postures and stretching combined with breathing regulation in this article. |
| Continuous Aerobic Exercise | Continuous aerobic exercise refers to rhythmic, cyclic physical activities performed over a sustained period, such as brisk walking, running, swimming, or cycling, which primarily target cardiovascular endurance and aerobic capacity. The concept specifically refers to moderate-intensity continuous aerobic sessions in this article. |
| Multicomponent Exercise | Multicomponent exercise is a training approach that integrates two or more modalities of physical activity to enhance overall fitness. The concept specifically refers to the combination of aerobic exercise and resistance training in this article. |
| QiGong | QiGong is a traditional Chinese mind-body practice that combines gentle physical movements, breathing techniques, and meditative concentration to promote relaxation, energy regulation (qi), and holistic health. The concept specifically refers to structured QiGong practice, including related forms such as Tai Chi, in this article. |
| High-Intensity Interval Training | High-intensity interval training (HIIT) is a workout method involving repeated bouts of short, vigorous exercise alternated with periods of active or passive recovery, designed to maximize cardiovascular and metabolic adaptations in a time-efficient manner. The concept specifically refers to structured HIIT sessions with predefined work-to-rest ratios in this article. |

**Supplementary Material S3: Kappa score for selecting studies between the reviewers.**

**Table S2.** Excluding studies by reading the title and abstract

| Reviewer 1 | Reviewer 2 | | | Total |
|---|---|---|---|---|
| | Exclude | Include | Unclear | |
| Exclude | 76 | 8 | 9 | 93 |
| Include | 5 | 156 | 5 | 166 |
| Unclear | 7 | 7 | 46 | 60 |
| Total | 88 | 171 | 60 | 319 |

Kappa score: 0.70 (0.60-0.80)

**Table S3.** Excluding studies by reading the full text

| Reviewer 1 | Reviewer 2 | | | Total |
|---|---|---|---|---|
| | Exclude | Include | Unclear | |
| Exclude | 72 | 1 | 6 | 79 |
| Include | 3 | 44 | 3 | 50 |
| Unclear | 1 | 2 | 21 | 27 |
| Total | 76 | 47 | 33 | 156 |

Kappa score: 0.80 (0.69-0.91)

**Supplementary Material S4: Dataset**

This supplementary file presents the datasets used in this study (i.e., exercise types and agent levels). *Study* indicates the type of disease. *Interventions* represents the types of exercise intervention. *Age* refers to the age of the patients, *year* indicates the publication year of the article, *Intervention duration* represents the total duration of exercise intervention (in weeks), *Frequency* indicates the frequency of intervention per week, *Time* represents the duration of each exercise session, *Exact dose* indicates the exact estimated metabolic equivalent (METs) accumulated by the participants each week during the study, *Study type* represents the design type of the clinical trial, *Measure* indicates the measurement method of the outcome indicators, and *Region* indicates the nationality of the authors.

| Study | Interventions/control, sample size (male) | Age (mean±sd) | Sample | Year | Intervention duration (weeks) | Frequency | Time /session | Exact dose | Dose | Residual dose | Study type | Measure | Region |
|---|---|---|---|---|---|---|---|---|---|---|---|---|---|
| Ee Suen Chan | qi gong | 23.5±0.00 | 18 | 2013 | 10 | 2 | 60 | 396 | 500 | -104 | Parallel-group | Salivary | Brunei |
| | placebo | 19.5±0.00 | 16 | | | | | 0 | 0 | 0 | RCT | | |
| Danucalov | yoga | 55.5±8.1 | 25 | 2013 | 8 | 3 | 75 | 517.5 | 500 | 17.5 | Parallel-group | Salivary | Brazil |
| | placebo | 53.4±8.2 | 21 | | | | | 0 | 0 | 0 | RCT | | |
| Rinske | yoga | 43.2±14.1 | 215 | 2017 | 12 | 7 | 15 | 241.5 | 250 | -8.5 | Parallel-group | Hair | Netherlands |
| | placebo | 43.2±13.7 | 109 | | | | | 0 | 0 | 0 | RCT | | |
| Lijun | continue aerobic exercise | 28.40±6.275 | 47 | 2022 | 8 | 3 | 40 | 576 | 500 | 76 | Parallel-group | Salivary | China |
| | placebo | 31.21±5.539 | 48 | | | | | 0 | 0 | 0 | RCT | | |
| Vadiraja | yoga | 46±9.13 | 14 | 2009 | 6 | 3 | 60 | 414 | 500 | -86 | Parallel-group | | India |
| | placebo | 48.45±10.21 | 11 | | | | | 0 | 0 | 0 | RCT | | |
| Chrysoula | continue aerobic exercise | 11.2 ±1.97 | 16 | 2018 | 8 | 7 | 60 | 2016 | 1000 | 1016 | Parallel-group | Salivary | Greece |
| | placebo | 11.2 ±1.97 | 20 | | | | | 0 | 0 | 0 | RCT | | |
| Putai | qi gong | 37.7± 14.3 | 11 | 1989 | 1 | 1 | 60 | 198 | 500 | -302 | Parallel-group | Salivary | Australia |
| | placebo | 33.2±9.0 | 11 | | | | | 0 | 0 | 0 | RCT | | |

| | | | | | | | | | | | | | |
|---|---|---|---|---|---|---|---|---|---|---|---|---|---|
| Marzieh | multicomponent exercise | 11.22 ± 1.90 | 20 | 2020 | 16 | 3 | 60 | 1260 | 1000 | 260 | Parallel-group | Serum | Iran |
| | placebo | 11.00 ± 2.67 | 20 | | | | | 0 | 0 | 0 | RCT | | |
| Mardia | stretch | 11.3±0.28 | 33 | 2020 | 8 | 1 | 120 | 276 | 250 | 26 | Parallel-group | Serum | Mexico |
| | placebo | 11.5±0.41 | 12 | | | | | 0 | 0 | 0 | RCT | | |
| Jean | multicomponent exercise | 9.3±2.0 | 44 | 2022 | 8 | 3 | 50 | 600 | 500 | 100 | Parallel-group | Salivary | USA |
| | placebo | 9.7±2.2 | 54 | | | | | 0 | 0 | 0 | RCT | | |
| Tiffany | yoga | 24.4 | 46 | 2013 | 12 | 7 | 20 | 322 | 250 | 72 | Parallel-group | Saliva | USA |
| | placebo | 24.5 | 46 | | | | | 0 | 0 | 0 | RCT | | |
| Jojo | yoga | 66.9±7.9 | 52 | 2025 | 8 | 1 | 90 | 180 | 250 | -70 | Parallel-group | Plasma | Hong Kong |
| | placebo | 63.3±7.5 | 54 | | | | | 0 | 0 | 0 | RCT | | |
| Nina | yoga | 37.27±11.85 | 22 | 2014 | 5 | 1 | 60 | 138 | 250 | -112 | Parallel-group | DEX/CRH | Germany |
| | placebo | 42.356±12.85 | 31 | | | | | 0 | 0 | 0 | RCT | | |
| Kyung | continue aerobic exercise | 75 | 44 | 2015 | 12 | 7 | 30 | 735 | 750 | -15 | Parallel-group | Blood | USA |
| | placebo | 72 | 24 | | | | | 0 | 0 | 0 | RCT | | |
| Madhuri | yoga | 36.94±8.94 | 29 | 2018 | 12 | 1 | 120 | 276 | 250 | 26 | Parallel-group | Blood | India |
| | placebo | 39.10±9.26 | 29 | | | | | 0 | 0 | 0 | RCT | | |
| Subbakrishna | yoga | 35.60±8.07 | 30 | 2006 | 2 | 4 | 30 | 276 | 250 | 26 | Parallel-group | Plasma | India |
| | placebo | 37.77±7.34 | 30 | | | | | 0 | 0 | 0 | RCT | | |
| Erin | qi gong | 70.14±7.77 | 14 | 2020 | 12 | 2 | 60 | 396 | 500 | -104 | Parallel-group | Plasma | Hong Kong |
| | placebo | 72.13±7.16 | 16 | | | | | 0 | 0 | 0 | RCT | | |
| Markus | continue aerobic exercise | 39.4±9.7 | 14 | 2020 | 6 | 3 | 50 | 1050 | 1000 | 50 | Parallel-group | Salivary | Switzerland |
| | stretch | 36.4±14.8 | 11 | | | | | 345 | 250 | 95 | RCT | | |
| Lara | multicomponent exercise | 41.5 | 9 | 2017 | 16 | 3 | 50 | 720 | 750 | -30 | Parallel-group | Plasma | Portugal |
| | placebo | 41.5 | 10 | | | | | 0 | 0 | 0 | RCT | | |
| Hector | qi gong | 79.67±6.55 | 14 | 2013 | 12 | 3 | 45 | 445.5 | 500 | -54.5 | Parallel-group | Salivary | Hong Kong |
| | placebo | 80.65±4.36 | 16 | | | | | 0 | 0 | 0 | RCT | | |

| | | | | | | | | | | | | | |
|---|---|---|---|---|---|---|---|---|---|---|---|---|---|
| Rainbow | continue aerobic exercise | 79.4±7.6 | 81 | 2018 | 12 | 2 | 60 | 420 | 500 | -80 | | Salivary | Hong Kong |
| | multicomponent exercise | 79.3±8.1 | 84 | | | | | 576 | 500 | 76 | Multi-arm | | |
| | placebo | 78.3±8.4 | 81 | | | | | 0 | 0 | 0 | RCT | | |
| Parisa | multicomponent exercise | 36.20±4.33 | 15 | 2023 | 8 | 3 | 70 | 588 | 500 | 88 | | Plasma | Malaysia |
| | yoga | 37.40±6.03 | 15 | | | | | 483 | 500 | -17 | Multi-arm | | |
| | placebo | 40.40±5.35 | 15 | | | | | 0 | 0 | 0 | RCT | | |
| Cherie | yoga | 34.17±15.75 | 18 | 2018 | 8 | 5 | 50 | 575 | 500 | 75 | | Salivary | Canada |
| | multicomponent exercise | 34.85±15.15 | 20 | | | | | 1825 | 1000 | 825 | Multi-arm | | |
| | placebo | 29.40±13.08 | 15 | | | | | 0 | 0 | 0 | RCT | | |
| Jolana | continue aerobic exercise | 58.8±7.1 | 10 | 2021 | 4 | 7 | 30 | 1218 | 1000 | 218 | | Hair | Austria |
| | stretch | 62.1±10.4 | 10 | | | | | 546 | 500 | 46 | Multi-arm | | |
| | yoga | 59.2±12.1 | 10 | | | | | 996 | 1000 | -4 | RCT | | |
| Elin | continue aerobic exercise | 35 | 40 | 2020 | 24 | 3 | 60 | 1314 | 1000 | 314 | Parallel-group | Plasma | Sweden |
| | placebo | 35 | 45 | | | | | 0 | 0 | 0 | RCT | | |
| Sudeep | yoga | 21.65±4.05 | 37 | 2023 | 12 | 5 | 60 | 690 | 750 | -60 | Parallel-group | Plasma | India |
| | placebo | 21.65±4.05 | 37 | | | | | 0 | 0 | 0 | RCT | | |
| Gopal | continue aerobic exercise | 22.8±1.6 | 20 | 2021 | 4 | 5 | 25 | 437.5 | 500 | -62.5 | Parallel-group | Plasma | Saudi Arabia |
| | placebo | 23.3±1.5 | 20 | | | | | 0 | 0 | 0 | RCT | | |
| Dimitris | HIIT | 42.4±12.7 | 10 | 2007 | 12 | 3 | 50 | 1095 | 1000 | 95 | Parallel-group | Plasma | Greece |
| | placebo | 41.5±12.9 | 10 | | | | | 0 | 0 | 0 | RCT | | |
| Hee | yoga | 67.00±9.13 | 21 | 2015 | 8 | 2 | 60 | 276 | 250 | 26 | | Plasma | Korea |
| | continue aerobic exercise | 63.33±8.83 | 18 | | | | | 516 | 500 | 16 | Multi-arm | | |
| | placebo | 68.47±6.17 | 17 | | | | | 0 | 0 | 0 | RCT | | |
| Bence | yoga | 24.7±3.9 | 34 | 2025 | 8 | 3 | 60 | 414 | 500 | -86 | Parallel-group | Salivary | Austria |
| | placebo | 25±4.5 | 39 | | | | | 0 | 0 | 0 | RCT | | |
| Franziska | continue aerobic exercise | 45±0.0 | 20 | 2017 | 2 | 4 | 50 | 960 | 1000 | -40 | Parallel-group | Salivary | Austria |

| | | | | | | | | | | | | | |
|---|---|---|---|---|---|---|---|---|---|---|---|---|---|
| | placebo | 45±0.0 | 21 | | | | | 0 | 0 | 0 | RCT | | |
| Dominika | HIIT | 31.11±4.03 | 35 | 2024 | 8 | 3 | 60 | 1260 | 1000 | 260 | Parallel-group | Hair | Poland |
| | placebo | 31.11±4.03 | 32 | | | | | 0 | 0 | 0 | RCT | | |
| Yuxia | qi gong | 57.7±8.3 | 25 | 2025 | 24 | 5 | 40 | 660 | 750 | -90 | Parallel-group | Serum | China |
| | placebo | 57.3±7.8 | 20 | | | | | 0 | 0 | 0 | RCT | | |
| Mohit | yoga | 40.0±12.9 | 7 | 2021 | 40 | 7 | 60 | 966 | 1000 | -34 | Parallel-group | Serum | India |
| | placebo | 38.7± 9.0 | 7 | | | | | 0 | 0 | 0 | RCT | | |
| Christine | HIIT | 59.4±4.9 | 195 | 2019 | 12 | 5 | 60 | 2100 | 1000 | 1100 | Parallel-group | Serum | Canada |
| | continue aerobic exercise | 59.6±5.1 | 191 | | | | | 720 | 750 | -30 | RCT | | |
| Lisa | qi gong | 54.33±3.55 | 9 | 2012 | 12 | 3 | 60 | 594 | 500 | 94 | Parallel-group | Serum | Usa |
| | placebo | 52.70±2.11 | 10 | | | | | 0 | 0 | 0 | RCT | | |
| Neha | yoga | 62.1±5.82 | 61 | 2016 | 8 | 3 | 60 | 414 | 250 | 164 | Parallel-group | Salivary | Usa |
| | placebo | 62.0±5.39 | 57 | | | | | 0 | 0 | 0 | RCT | | |
| Maheshkuma | yoga | 13.76±0.45 | 13 | 2021 | 24 | 3 | 45 | 310 | 250 | 60 | Parallel-group | Salivary | India |
| | placebo | 14.46±0.87 | 13 | | | | | 0 | 0 | 0 | RCT | | |
| Claudia | placebo | 51.20±10.52 | 17 | | | | | 0 | 0 | 0 | | | |
| | continue aerobic exercise | 51.67±10.68 | 12 | 2024 | 16 | 4 | 50 | 960 | 1000 | -40 | Multi-arm | Blood | Italy |
| | HIIT | 51.81±10.39 | 13 | | | | | 630 | 750 | -120 | RCT | | |
| Shreen | continue aerobic exercise | 27.23±3.73 | 30 | 2025 | 12 | 5 | 50 | 750 | 750 | 0 | Parallel-group | Serum | Egypt |
| | placebo | 26.90±4.05 | 30 | | | | | 0 | 0 | 0 | RCT | | |
| Muzeyyen | multicomponent exercise | 38 | 18 | 2024 | 6 | 3 | 60 | 720 | 750 | -30 | Parallel-group | Serum | |
| | yoga | 41 | 18 | | | | | 414 | 500 | -86 | RCT | | |
| Elin | continue aerobic exercise | 10.4±1.37 | 59 | 2022 | 32 | 2 | 60 | 360 | 250 | 110 | Parallel-group | Salivary | Sweden |
| | placebo | 10.7±1.32 | 55 | | | | | 0 | 0 | 0 | RCT | | |
| Abhishek | yoga | 44 | 111 | 2016 | 12 | 7 | 45 | 724.5 | 750 | -25.5 | Parallel-group | Serum | India |
| | multicomponent exercise | 44 | 105 | | | | | 630 | 750 | -120 | RCT | | |

| | | | | | | | | | | | | | |
|---|---|---|---|---|---|---|---|---|---|---|---|---|---|
| Naveen | yoga | 28.07±2.60 | 35 | 2024 | 8 | 5 | 60 | 690 | 750 | -60 | Parallel-group | Serum | India |
| | placebo | 28.02±2.79 | 37 | | | | | 0 | 0 | 0 | RCT | | |

## Supplementary Material S5: Network meta- analysis supplementary

## S5.1 Details of pairwise meta-analyses

**Table S4.** Details of pairwise meta-analyses

| Comparison | Number of studies | MD | 95%CrI | $I^2$ |
|---|---|---|---|---|
| CAE vs CG | 37 | **-0.05** | **(-0.44, 0.34)** | 84.92% |
| QG vs CG | 6 | **-0.42** | **(-1.32, 0.48)** | 87.62% |
| HIIT vs CG | 3 | **0.53** | **(0.09, 0.97)** | 23.46% |
| MCE vs CG | 6 | **-0.01** | **(-0.21, 0.19)** | 0.00% |
| Yoga vs CG | 17 | **-0.67** | **(-1.08, -0.25)** | 90.86% |

## S5.2 Comparison funnel plot of all studies

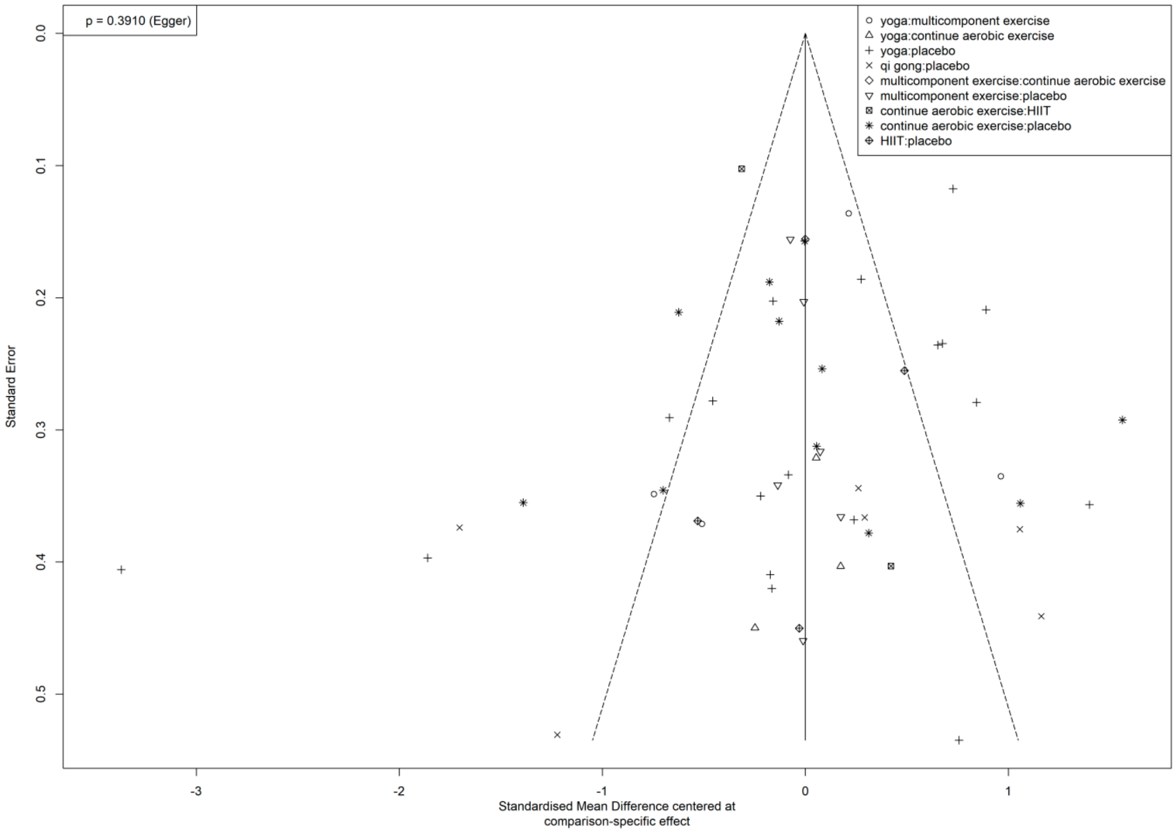


**Figure S1.** Funnel plot of all studies

### S5.3 Dot splitting method to explore inconsistency

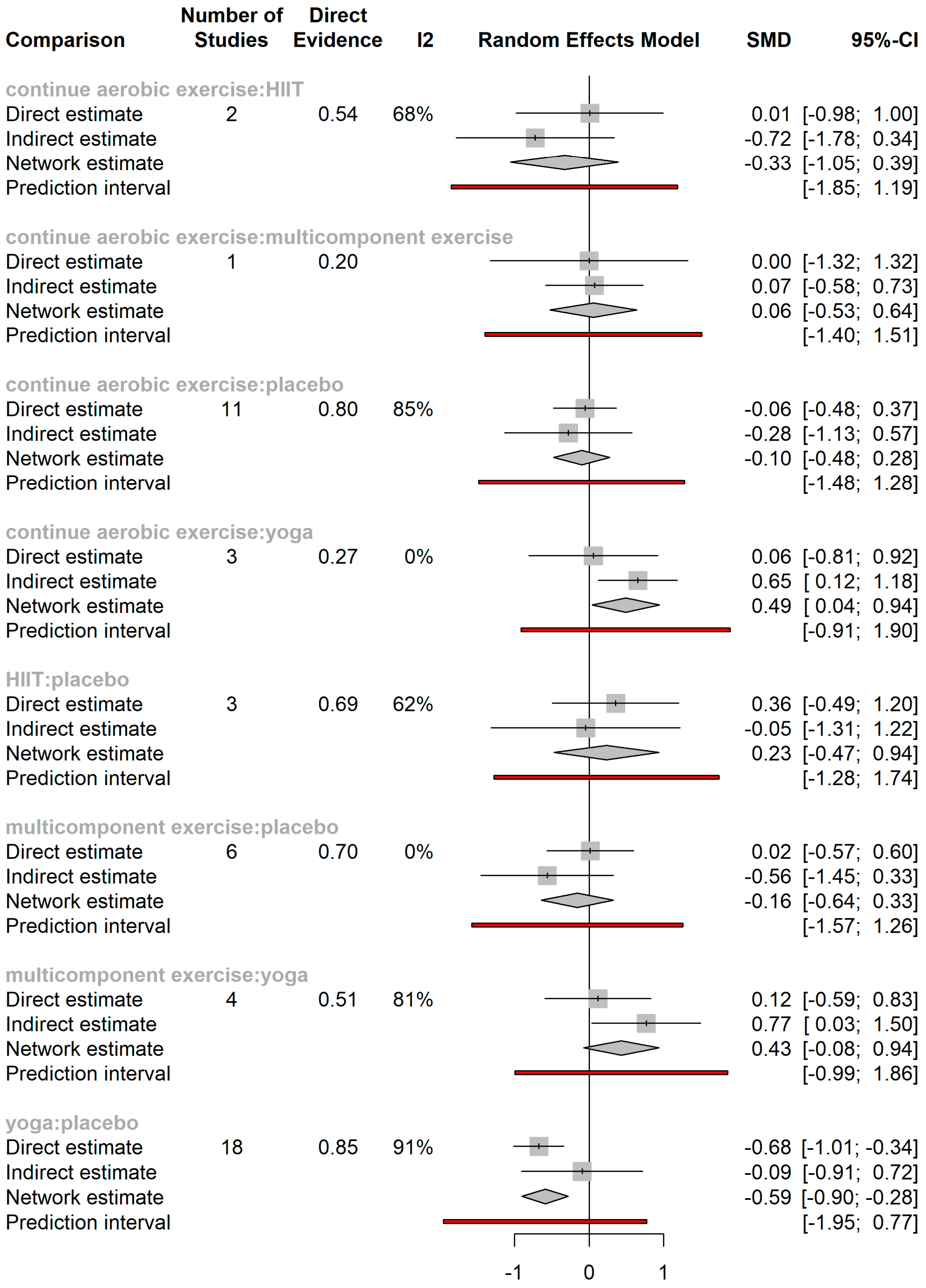


**Figure S2.** Dot splitting method to explore inconsistency. I2 represents the heterogeneity of pairwise comparisons and the red region represents the prediction interval.

## S5.4 Rank

### S5.4.1 SUCRA table for all studies

**Table S5.** SUCRA table for all studies

| Treatment | Rank1 | Rank2 | Rank3 | Rank4 | Rank5 | Rank6 | Sucra |
|---|---|---|---|---|---|---|---|
| Plocle | 0.00 | 0.11 | 0.30 | 0.30 | 0.18 | 0.08 | 0.04 |
| CAE | 0.00 | 0.14 | 0.09 | 0.29 | 0.47 | 0.12 | 0.42 |
| HIIT | 0.00 | 0.04 | 0.08 | 0.09 | 0.10 | 0.66 | 0.85 |
| MCE | 0.03 | 0.17 | 0.32 | 0.21 | 0.16 | 0.08 | 0.51 |
| QG | 0.28 | 0.36 | 0.15 | 0.08 | 0.07 | 0.03 | 0.68 |
| Yoga | 0.66 | 0.29 | 0.04 | 0.00 | 0.00 | 0.00 | |

### S5.4.2 Heat map

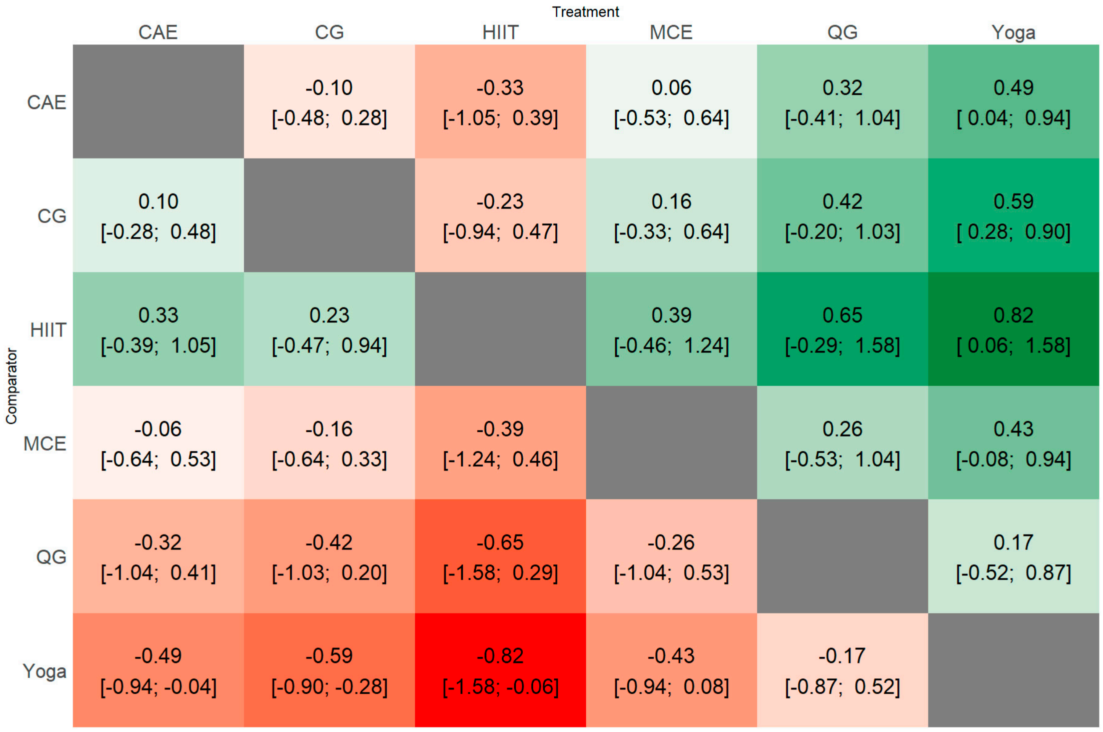


**Figure S3**. Heat map. It ranks the effectiveness of exercise intervention measures for patients with psychological distress disorders. The numbers represent the SUCRA scoring system, which divides exercise intensity into continuous levels ranging from 0 to 1.

## S5.5 GRADE Assessment

**Table S6.** GRADE assessment for all pairwise comparisons

| Comparisons | Nature of evidence | Certainty | Reason for downgrade |
|---|---|---|---|
| CAE vs. HIIT | Direct | Low | Imprecision, indirectness |
| CAE vs. MCE | Direct | Low | Imprecision, indirectness |
| CAE vs. Control | Direct | Low | Imprecision, indirectness |
| CAE vs. Yoga | Direct | Low | Imprecision, indirectness |
| CAE vs. Qigong | Indirect | Low | Imprecision, indirectness |
| HIIT vs. MCE | Mixed | Low | Imprecision |
| HIIT vs. Control | Direct | Low | Imprecision, indirectness |
| HIIT vs. Yoga | Mixed | Low | Imprecision, risk of bias |
| HIIT vs. Qigong | Indirect | Low | Imprecision, indirectness |
| MCE vs. Control | Direct | Low | Imprecision, indirectness |
| MCE vs. Yoga | Direct | Low | Imprecision, indirectness |
| MCE vs. Qigong | Indirect | Low | Imprecision, indirectness |
| Yoga vs. Control | Direct | High | No downgrade |
| Yoga vs. Qigong | Indirect | Low | Imprecision, indirectness |
| Qigong vs. Control | Direct | Low | Imprecision, indirectness |

## S5.6 Meta-regression

**Table S7.** Model fit summaries for univariate network meta-regression.

| Covariate | DIC | pD | Residual Deviance | Shared beta (Median and 95% CrI) | SD |
|---|---|---|---|---|---|
| year | 185.4 | 92.0 | 93.4 | -159.6 (-806.4 , 408.2) | 127.7(29.2, 200.8) |
| age | 183.4 | 90.6 | 92.7 | 95.8(-134.2, 699.4) | 90.4(39.5,200.7) |
| weeks | 202.1 | 98.5 | 103.5 | -100.7(-555.7, 311.7) | 107.3(10.5, 200.4) |

*Note*. The network meta-regression model was fitted in a Bayesian framework using the Markov chain Monte Carlo (MCMC) method in the R statistical package "Gemtc". Four chains were run using a non-informative prior. The number of iterations for each chain was 25,000, with the first 5,000 iterations discarded.

## Supplementary Material S6: Dose network meta- analysis supplementary

### S6.1:Key assumptions of Network Meta-Analysis

There are three key assumptions to conduct a Network Meta-Analysis (NMA): (1) network connectivity, (2) consistency in the data, and (3) transitivity.

### S6.1.1:Connectivity

Connectivity is a key assumption in NMA which if deemed insufficient (i.e., due to lack of direct comparators) can lead to low statistical power and misleading results[3]. In our study, we assessed connectivity of the network at both treatment and agent levels visually and found no evidence of unconnectedness on either network (Supplementary Figure S1 and Supplementary Figure S2).

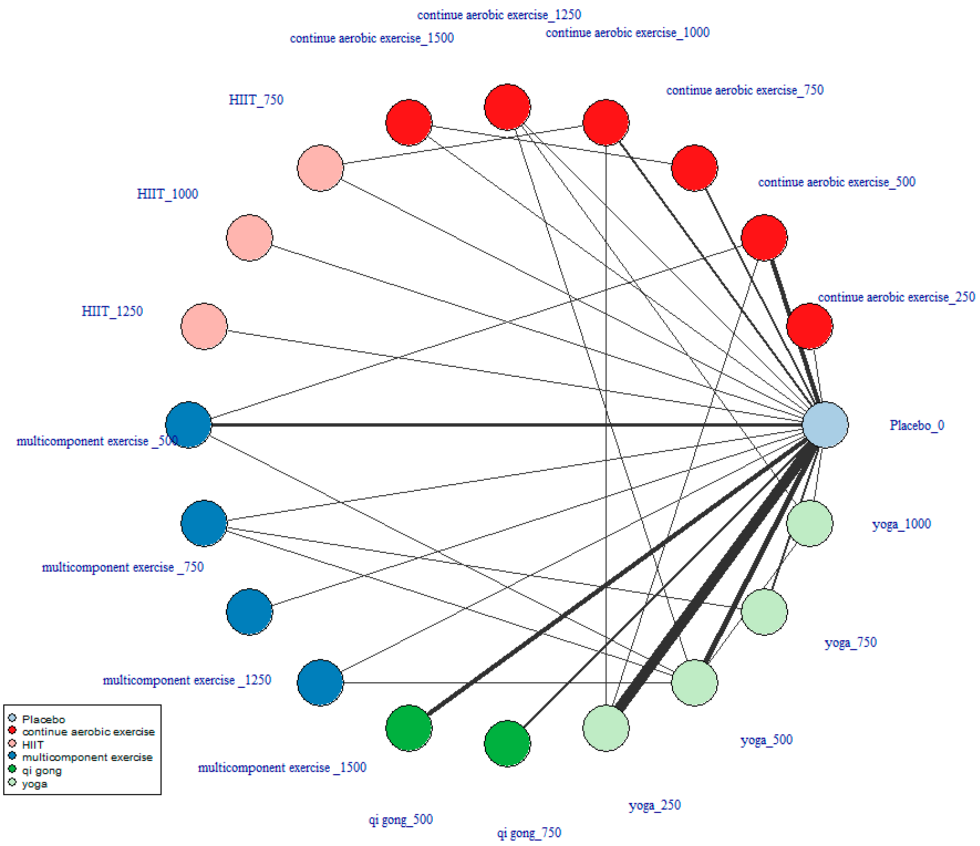


**Figure S4**.Treatment-level network. The first part of the word represents the intervention, and the second part represents the corresponding dose.

**S6.1.2:Consistency**

**Table S8.** Consistent and UME models fit comparison

| Model | pD | Total Residual deviance | DIC | SD |
|---|---|---|---|---|
| Consistent | 86.20 | 41.53 | 127.00 | 0.53 |
| UME | 88.6 | 42.08 | 130.20 | 0.52 |

*Note.* pD: Number of estimated parameters; DIC: Deviance Informative Criterion; SD: Standard
Deviation; UME: Unrelated Mean Effects. Scientific literature indicated that the main indicator to
assess the model fit is the DIC. As lower DIC, better fit.

### S6.1.3:Transitivity

**Table S9.** Comparison of transitivity

| Comparison | p-value | Median | 2.5% | 97.5% |
|---|---|---|---|---|

| | | | | |
|---|---|---|---|---|
| yoga_750 vs multicomponent exercise _750 | 0.472 | | | |
| -> direct | | 0.127 | -1.971 | 2.256 |
| -> indirect | | 0.986 | 0.098 | 1.931 |
| -> MBNMA | | 0.844 | -0.037 | 1.683 |
| yoga_500 vs multicomponent exercise _750 | 0.504 | | | |
| -> direct | | 0.421 | -1.948 | 2.597 |
| -> indirect | | 0.433 | -0.306 | 1.134 |
| -> MBNMA | | 0.420 | -0.322 | 1.124 |
| yoga_1000 vs continue aerobic exercise_1250 | 0.213 | | | |
| -> direct | | -0.142 | -2.264 | 2.054 |
| -> indirect | | 1.929 | 0.726 | 3.096 |
| -> MBNMA | | 1.534 | 0.382 | 2.672 |
| yoga_500 vs continue aerobic exercise_1250 | 0.556 | | | |
| -> direct | | 0.427 | -1.930 | 2.891 |
| -> indirect | | 0.731 | -0.223 | 1.661 |
| -> MBNMA | | 0.687 | -0.185 | 1.552 |
| yoga_250 vs continue aerobic exercise_1000 | 0.482 | | | |
| -> direct | | 0.217 | -2.011 | 2.538 |
| -> indirect | | 0.292 | -0.385 | 0.967 |
| -> MBNMA | | 0.295 | -0.360 | 0.943 |
| continue aerobic exercise_1500 vs continue aerobic exercise_750 | 0.407 | | | |
| -> direct | | 0.390 | -1.648 | 2.450 |
| -> indirect | | 0.085 | -0.431 | 0.568 |
| -> MBNMA | | 0.103 | -0.397 | 0.604 |
| qi gong_750 vs Placebo_0 | 0.413 | | | |
| -> direct | | 1.411 | -0.243 | 3.016 |
| -> indirect | | -0.029 | -1.770 | 1.679 |
| -> MBNMA | | 0.713 | -0.473 | 1.941 |
| qi gong_500 vs Placebo_0 | 0.427 | | | |
| -> direct | | 0.004 | -1.181 | 1.115 |
| -> indirect | | 0.911 | -0.194 | 2.067 |

| | | | | |
|---|---|---|---|---|
| -> MBNMA | | 0.475 | -0.315 | 1.294 |
| multicomponent exercise _1500 vs Placebo_0 | 0.430 | | | |
| -> direct | | -0.153 | -2.399 | 2.072 |
| -> indirect | | 1.363 | -0.246 | 2.876 |
| -> MBNMA | | 0.865 | -0.439 | 2.189 |
| multicomponent exercise _1250 vs Placebo_0 | 0.648 | | | |
| -> direct | | 0.273 | -1.929 | 2.529 |
| -> indirect | | 0.857 | -0.371 | 2.092 |
| -> MBNMA | | 0.720 | -0.365 | 1.824 |
| multicomponent exercise _500 vs Placebo_0 | 0.589 | | | |
| -> direct | | 0.220 | -0.995 | 1.469 |
| -> indirect | | 0.300 | -0.171 | 0.777 |
| -> MBNMA | | 0.288 | -0.146 | 0.730 |
| HIIT_1250 vs Placebo_0 | 0.660 | | | |
| -> direct | | -1.113 | -3.350 | 0.925 |
| -> indirect | | -0.129 | -2.411 | 2.269 |
| -> MBNMA | | -0.627 | -2.212 | 0.904 |
| HIIT_1000 vs Placebo_0 | 0.722 | | | |
| -> direct | | -0.232 | -2.720 | 2.093 |
| -> indirect | | -0.600 | -2.020 | 0.835 |
| -> MBNMA | | -0.501 | -1.770 | 0.723 |
| HIIT_750 vs Placebo_0 | 0.599 | | | |
| -> direct | | 0.076 | -1.980 | 2.189 |
| -> indirect | | -0.504 | -1.500 | 0.539 |
| -> MBNMA | | -0.376 | -1.327 | 0.542 |
| continue aerobic exercise_500 vs Placebo_0 | 0.201 | | | |
| -> direct | | 0.819 | -0.211 | 1.896 |
| -> indirect | | -0.005 | -0.343 | 0.338 |
| -> MBNMA | | 0.069 | -0.264 | 0.402 |
| continue aerobic exercise_250 vs Placebo_0 | 0.163 | | | |
| -> direct | | 0.377 | -1.711 | 2.427 |

| | | | |
|---|---|---|---|
| -> indirect | 0.032 | -0.125 | 0.199 |
| -> MBNMA | 0.034 | -0.132 | 0.201 |

## S6.2 Models' selection

**S6.2.1.** Nonlinear functions and models fit comparison

A meta-analysis (i.e., a "split" NMA) of the different doses of physical activity as separate and unrelated treatments were performed. This step helps determine which function is more appropriate for the data and should be used in a model-based network meta-analysis (MBNMA). **Figure S5** and **Figure S6** show the different responses of each dose to overall and different types of exercise, respectively (Hedges' g).

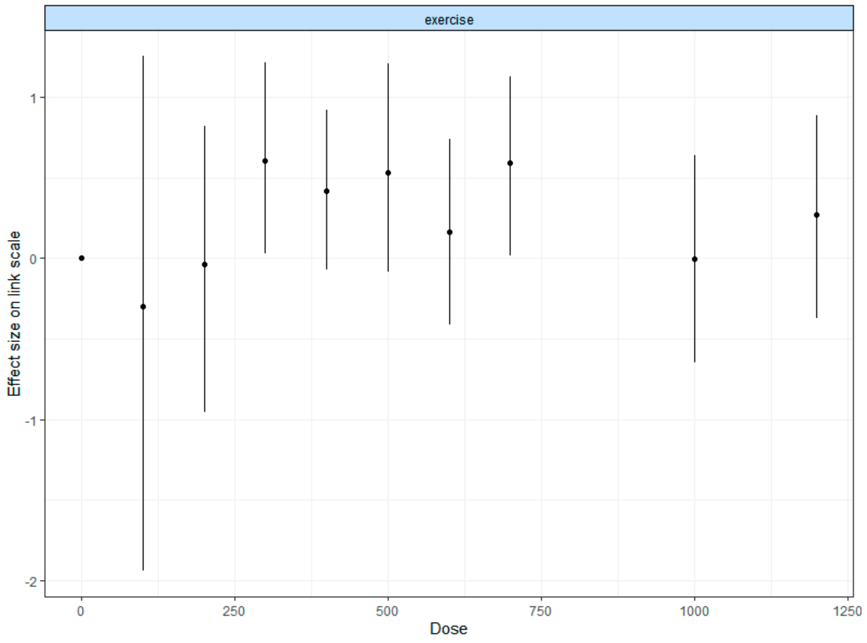


**Figure S5 .** "Split" NMA of overall exercise

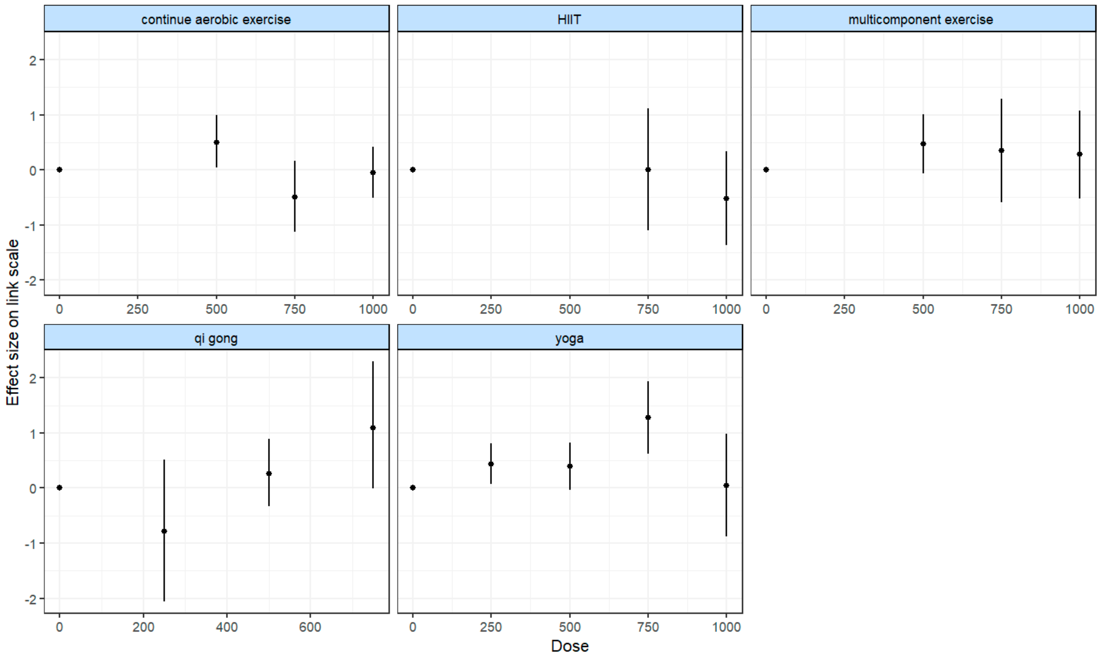


**Figure S6.** "Split" NMA of different exercise agents.

**Table S10.** Models Fit Comparison For Overall Exercise

| Model | DIC | SD | Deviance | Total Residual Deviance | pD |
|---|---|---|---|---|---|
| Restricted cubic spline (common treatment effects; 3 knots) | 319.8 | NA | 272.829 | 324.521 | 47.8 |
| Restricted cubic spline (random treatment effects; 3 knots) | 123.0 | 0.581 | 40.349 | 92.04 | 83.3 |
| Polynomial (common treatment effects) | 319.4 | NA | 274.533 | 326.225 | 45.6 |
| Non-parametric monotonically up (common treatment effects) | 317.7 | NA | 272.854 | 324.546 | 45.6 |
| Non-parametric monotonically up (random treatment effects) | 122.8 | 0.576 | 40.287 | 91.979 | 83.0 |

**Table S11.** Models fit Comparison For Different Type Exercise

| Model | DIC | SD | Deviance | Total Residual Deviance | pD |
|---|---|---|---|---|---|
| Restricted cubic spline (common treatment effects; 3 knots) | 237.8 | NA | 179.912 | 231.604 | 58.6 |
| Restricted cubic spline (random treatment effects; 3 knots) | 125.2 | 0.527 | 41.124 | 92.816 | 84.6 |
| Polynomial (common treatment effects) | 276.6 | NA | 224.785 | 276.477 | 52.5 |
| Non-parametric monotonically up (common treatment effects) | 273.9 | NA | 218.313 | 270.005 | 56.2 |
| Non-parametric monotonically up (random treatment effects) | 126.7 | 0.572 | 41.329 | 93.021 | 85.9 |

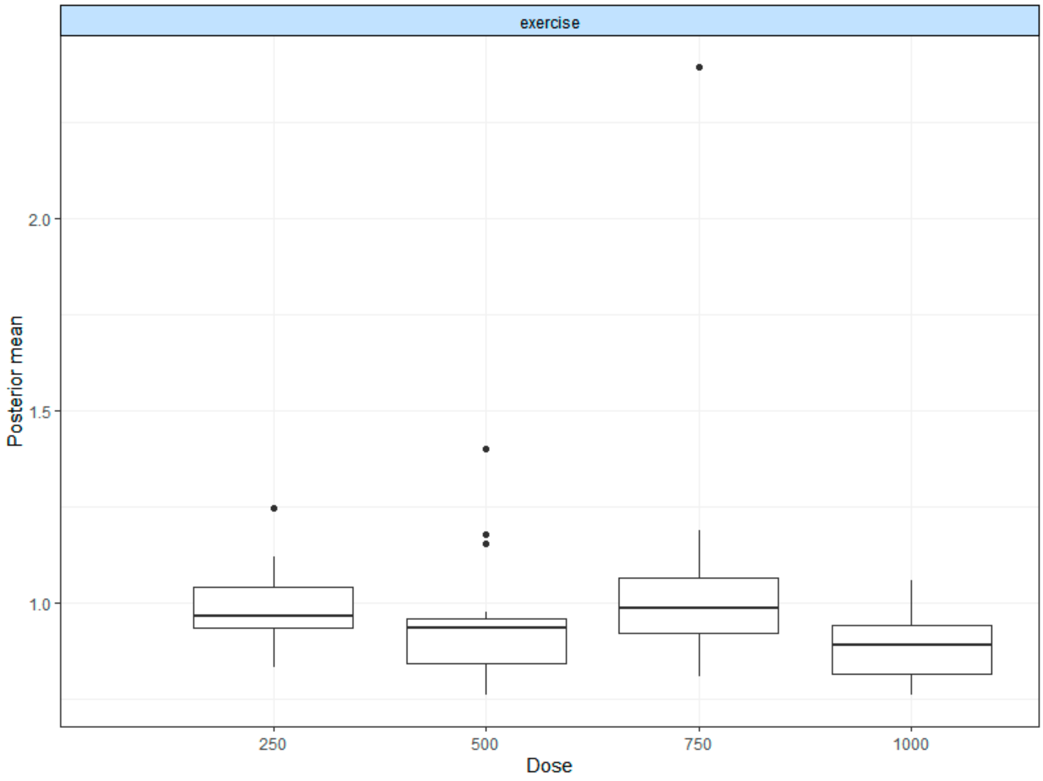


**Figure S7.** Deviance plot at overall exercise level.

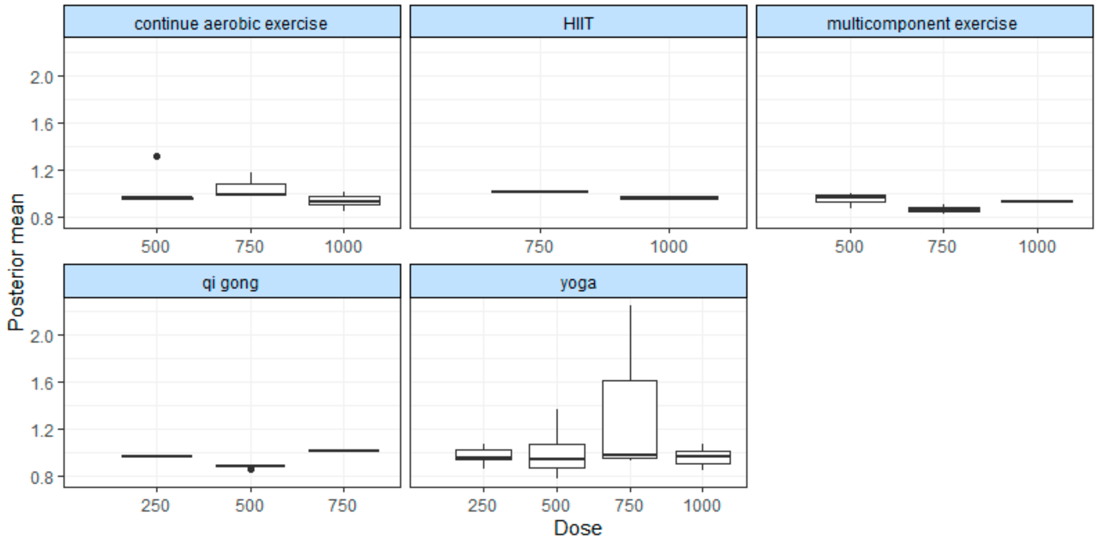


**Figure S8.** Deviance plots at treatment-level.

## Supplementary Material S7:Ranking of effectiveness of interventions

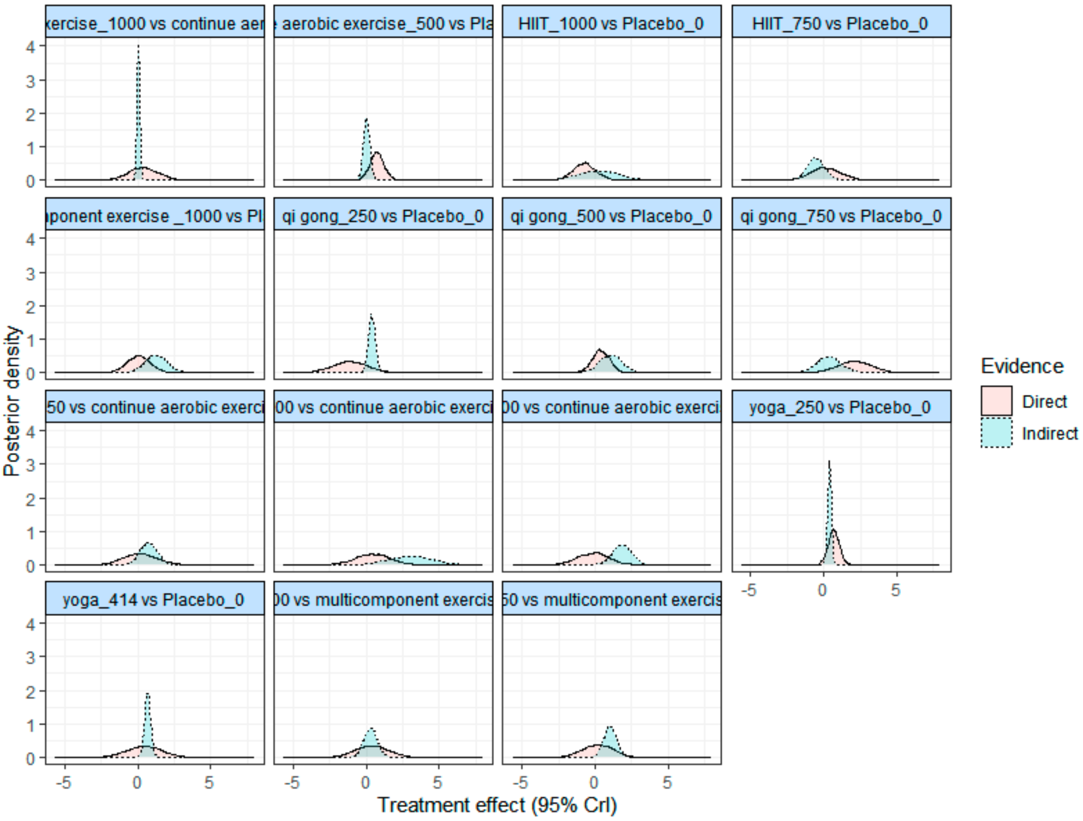


**Figure S9.** Node-splitting analysis (density plot).

## Supplementary Material S8: Subgroup analysis

**A**

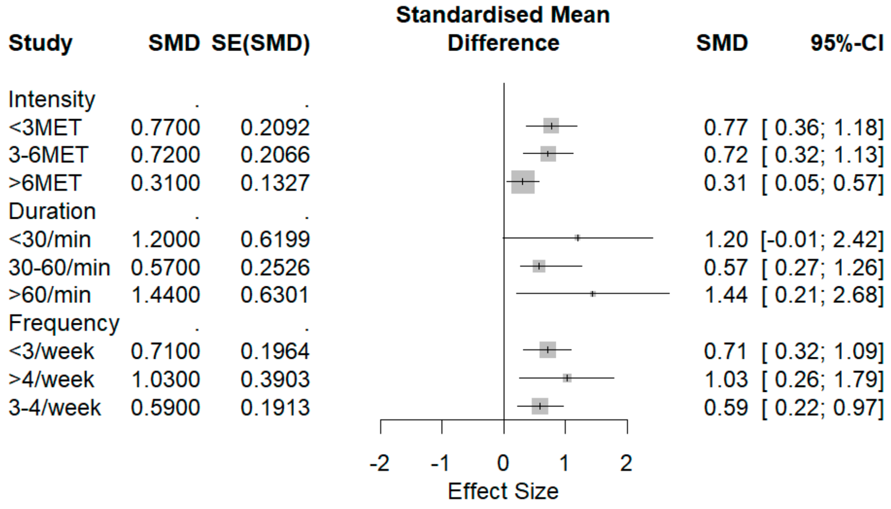


**B**

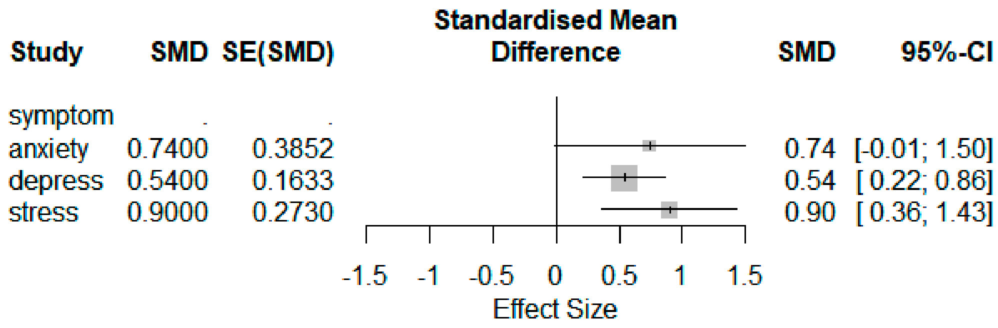


**C**

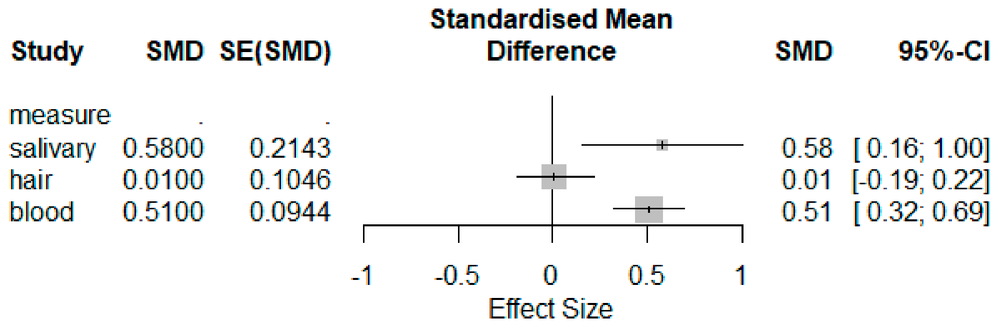


**Figure S10. Subgroup analysis**

*Note.*The three panels present the following subgroup analyses: (A) Exercise parameters, including intensity, frequency, and duration; (B) Primary symptoms of psychological distress assessed in this study, including anxiety, depression, and stress; (C) Biomarker sample types used for cortisol measurement, including saliva, hair, and blood.

## Supplementary Material S9: Risk bias Analysis

**A**

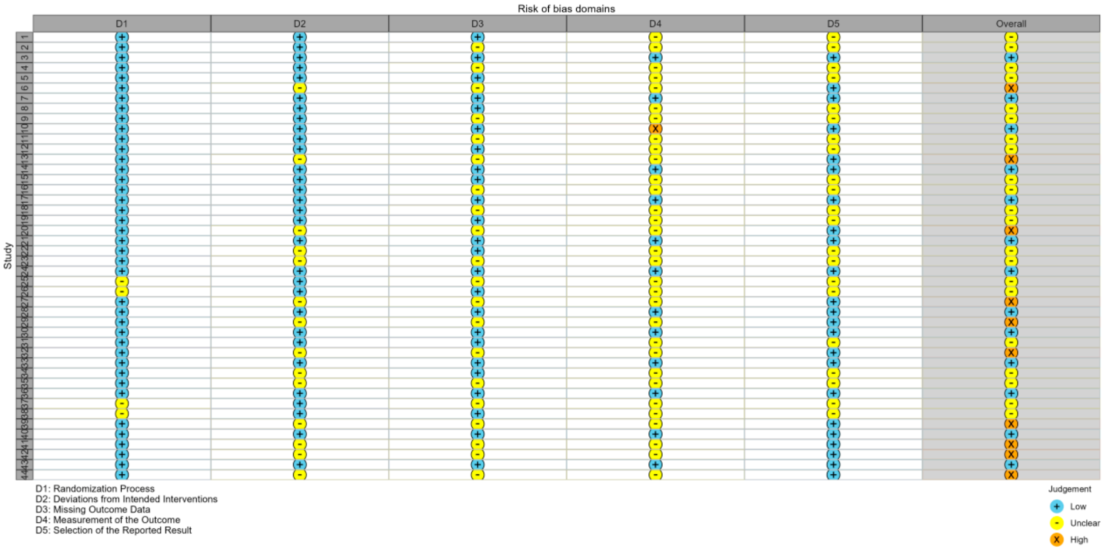


B

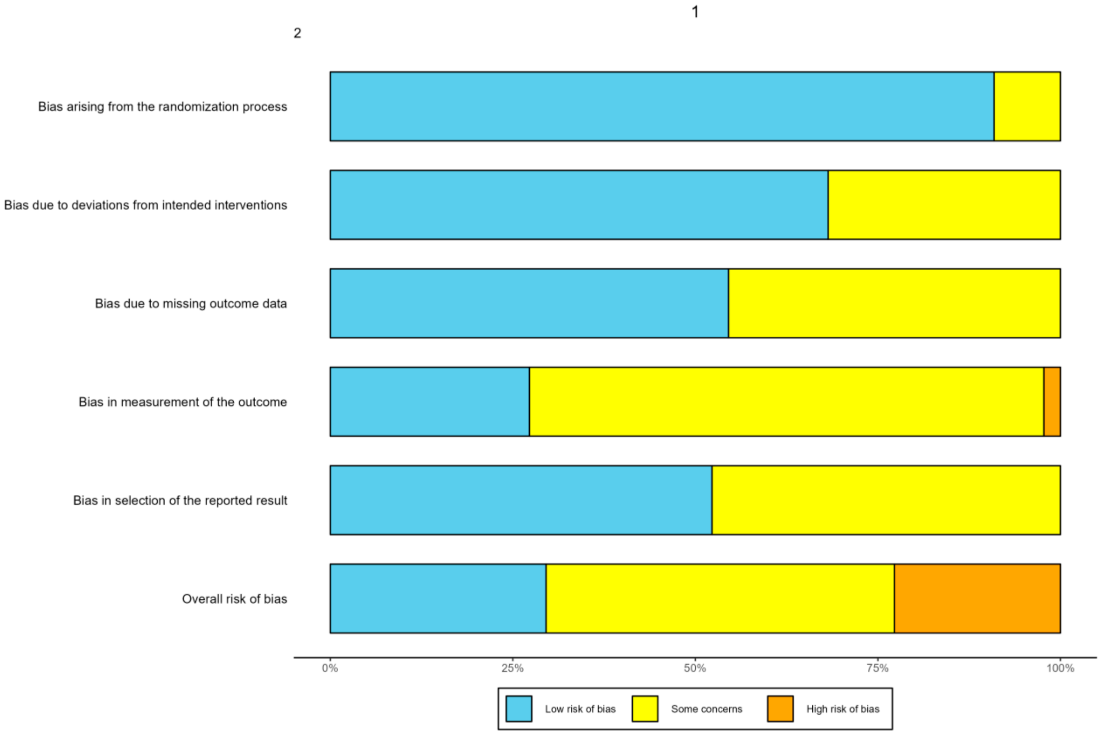


**FigS11. Study-level of Risk bias Analysis**

*Note.* A : Study characteristics and assessment of risk bias; B : Summary of risk bias across studies

**Supplementary Material S10: Sensitivity analysis including only studies with low**

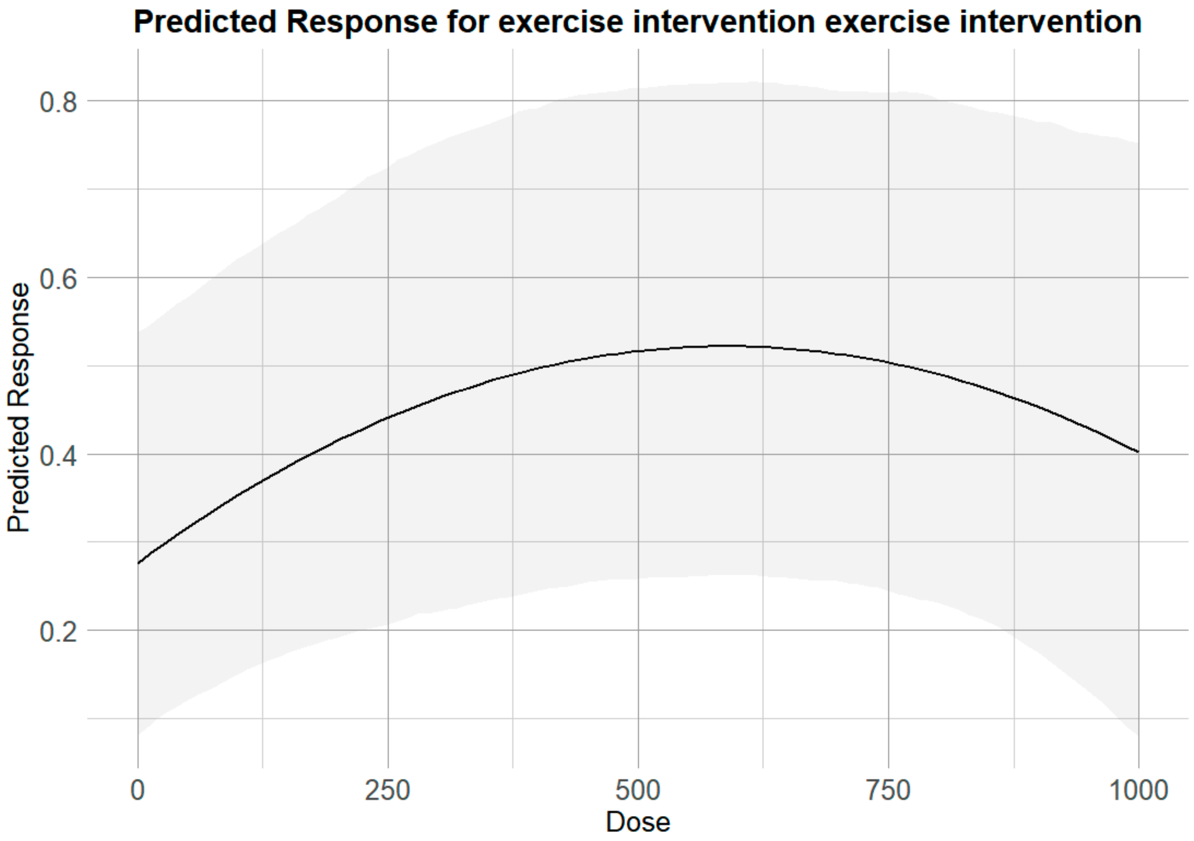


**Figure S12.** Dose-response curve between exercise and improves in psychological distress only including studies with low risk of bias.

## Supplementary Material S11:Ranking of effectiveness of interventions

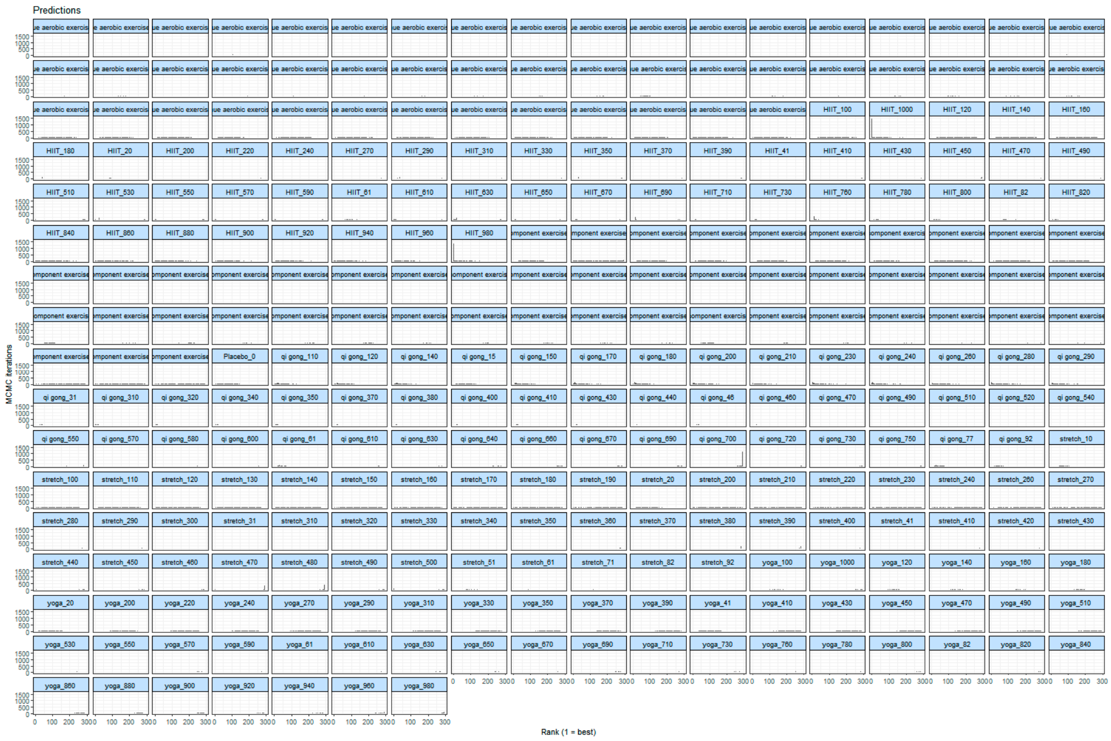

**Figure S13.** Effectiveness ranking by exercise treatments
